# Measurement of the relative prompt production rate of *χ*_c2_ and *χ*_c1_ in pp collisions at $\sqrt{s} = 7\ \mathrm{TeV}$

**DOI:** 10.1140/epjc/s10052-012-2251-3

**Published:** 2012-12-14

**Authors:** The CMS Collaboration, S. Chatrchyan, V. Khachatryan, A. M. Sirunyan, A. Tumasyan, W. Adam, E. Aguilo, T. Bergauer, M. Dragicevic, J. Erö, C. Fabjan, M. Friedl, R. Frühwirth, V. M. Ghete, J. Hammer, N. Hörmann, J. Hrubec, M. Jeitler, W. Kiesenhofer, V. Knünz, M. Krammer, I. Krätschmer, D. Liko, I. Mikulec, M. Pernicka, B. Rahbaran, C. Rohringer, H. Rohringer, R. Schöfbeck, J. Strauss, A. Taurok, W. Waltenberger, G. Walzel, E. Widl, C.-E. Wulz, V. Mossolov, N. Shumeiko, J. Suarez Gonzalez, M. Bansal, S. Bansal, T. Cornelis, E. A. De Wolf, X. Janssen, S. Luyckx, L. Mucibello, S. Ochesanu, B. Roland, R. Rougny, M. Selvaggi, Z. Staykova, H. Van Haevermaet, P. Van Mechelen, N. Van Remortel, A. Van Spilbeeck, F. Blekman, S. Blyweert, J. D’Hondt, R. Gonzalez Suarez, A. Kalogeropoulos, M. Maes, A. Olbrechts, W. Van Doninck, P. Van Mulders, G. P. Van Onsem, I. Villella, B. Clerbaux, G. De Lentdecker, V. Dero, A. P. R. Gay, T. Hreus, A. Léonard, P. E. Marage, A. Mohammadi, T. Reis, L. Thomas, G. Vander Marcken, C. Vander Velde, P. Vanlaer, J. Wang, V. Adler, K. Beernaert, A. Cimmino, S. Costantini, G. Garcia, M. Grunewald, B. Klein, J. Lellouch, A. Marinov, J. Mccartin, A. A. Ocampo Rios, D. Ryckbosch, N. Strobbe, F. Thyssen, M. Tytgat, P. Verwilligen, S. Walsh, E. Yazgan, N. Zaganidis, S. Basegmez, G. Bruno, R. Castello, L. Ceard, C. Delaere, T. du Pree, D. Favart, L. Forthomme, A. Giammanco, J. Hollar, V. Lemaitre, J. Liao, O. Militaru, C. Nuttens, D. Pagano, A. Pin, K. Piotrzkowski, N. Schul, J. M. Vizan Garcia, N. Beliy, T. Caebergs, E. Daubie, G. H. Hammad, G. A. Alves, M. Correa Martins Junior, D. De Jesus Damiao, T. Martins, M. E. Pol, M. H. G. Souza, W. L. Aldá Júnior, W. Carvalho, A. Custódio, E. M. Da Costa, C. De Oliveira Martins, S. Fonseca De Souza, D. Matos Figueiredo, L. Mundim, H. Nogima, V. Oguri, W. L. Prado Da Silva, A. Santoro, L. Soares Jorge, A. Sznajder, T. S. Anjos, C. A. Bernardes, F. A. Dias, T. R. Fernandez Perez Tomei, E. M. Gregores, C. Lagana, F. Marinho, P. G. Mercadante, S. F. Novaes, Sandra S. Padula, V. Genchev, P. Iaydjiev, S. Piperov, M. Rodozov, S. Stoykova, G. Sultanov, V. Tcholakov, R. Trayanov, M. Vutova, A. Dimitrov, R. Hadjiiska, V. Kozhuharov, L. Litov, B. Pavlov, P. Petkov, J. G. Bian, G. M. Chen, H. S. Chen, C. H. Jiang, D. Liang, S. Liang, X. Meng, J. Tao, J. Wang, X. Wang, Z. Wang, H. Xiao, M. Xu, J. Zang, Z. Zhang, C. Asawatangtrakuldee, Y. Ban, Y. Guo, W. Li, S. Liu, Y. Mao, S. J. Qian, H. Teng, D. Wang, L. Zhang, W. Zou, C. Avila, J. P. Gomez, B. Gomez Moreno, A. F. Osorio Oliveros, J. C. Sanabria, N. Godinovic, D. Lelas, R. Plestina, D. Polic, I. Puljak, Z. Antunovic, M. Kovac, V. Brigljevic, S. Duric, K. Kadija, J. Luetic, S. Morovic, A. Attikis, M. Galanti, G. Mavromanolakis, J. Mousa, C. Nicolaou, F. Ptochos, P. A. Razis, M. Finger, M. Finger, Y. Assran, S. Elgammal, A. Ellithi Kamel, S. Khalil, M. A. Mahmoud, A. Radi, M. Kadastik, M. Müntel, M. Raidal, L. Rebane, A. Tiko, P. Eerola, G. Fedi, M. Voutilainen, J. Härkönen, A. Heikkinen, V. Karimäki, R. Kinnunen, M. J. Kortelainen, T. Lampén, K. Lassila-Perini, S. Lehti, T. Lindén, P. Luukka, T. Mäenpää, T. Peltola, E. Tuominen, J. Tuominiemi, E. Tuovinen, D. Ungaro, L. Wendland, K. Banzuzi, A. Karjalainen, A. Korpela, T. Tuuva, M. Besancon, S. Choudhury, M. Dejardin, D. Denegri, B. Fabbro, J. L. Faure, F. Ferri, S. Ganjour, A. Givernaud, P. Gras, G. Hamel de Monchenault, P. Jarry, E. Locci, J. Malcles, L. Millischer, A. Nayak, J. Rander, A. Rosowsky, I. Shreyber, M. Titov, S. Baffioni, F. Beaudette, L. Benhabib, L. Bianchini, M. Bluj, C. Broutin, P. Busson, C. Charlot, N. Daci, T. Dahms, L. Dobrzynski, R. Granier de Cassagnac, M. Haguenauer, P. Miné, C. Mironov, I. N. Naranjo, M. Nguyen, C. Ochando, P. Paganini, D. Sabes, R. Salerno, Y. Sirois, C. Veelken, A. Zabi, J.-L. Agram, J. Andrea, D. Bloch, D. Bodin, J.-M. Brom, M. Cardaci, E. C. Chabert, C. Collard, E. Conte, F. Drouhin, C. Ferro, J.-C. Fontaine, D. Gelé, U. Goerlach, P. Juillot, A.-C. Le Bihan, P. Van Hove, F. Fassi, D. Mercier, S. Beauceron, N. Beaupere, O. Bondu, G. Boudoul, J. Chasserat, R. Chierici, D. Contardo, P. Depasse, H. El Mamouni, J. Fay, S. Gascon, M. Gouzevitch, B. Ille, T. Kurca, M. Lethuillier, L. Mirabito, S. Perries, V. Sordini, Y. Tschudi, P. Verdier, S. Viret, Z. Tsamalaidze, G. Anagnostou, C. Autermann, S. Beranek, M. Edelhoff, L. Feld, N. Heracleous, O. Hindrichs, R. Jussen, K. Klein, J. Merz, A. Ostapchuk, A. Perieanu, F. Raupach, J. Sammet, S. Schael, D. Sprenger, H. Weber, B. Wittmer, V. Zhukov, M. Ata, J. Caudron, E. Dietz-Laursonn, D. Duchardt, M. Erdmann, R. Fischer, A. Güth, T. Hebbeker, C. Heidemann, K. Hoepfner, D. Klingebiel, P. Kreuzer, C. Magass, M. Merschmeyer, A. Meyer, M. Olschewski, P. Papacz, H. Pieta, H. Reithler, S. A. Schmitz, L. Sonnenschein, J. Steggemann, D. Teyssier, M. Weber, M. Bontenackels, V. Cherepanov, Y. Erdogan, G. Flügge, H. Geenen, M. Geisler, W. Haj Ahmad, F. Hoehle, B. Kargoll, T. Kress, Y. Kuessel, A. Nowack, L. Perchalla, O. Pooth, P. Sauerland, A. Stahl, M. Aldaya Martin, J. Behr, W. Behrenhoff, U. Behrens, M. Bergholz, A. Bethani, K. Borras, A. Burgmeier, A. Cakir, L. Calligaris, A. Campbell, E. Castro, F. Costanza, D. Dammann, C. Diez Pardos, G. Eckerlin, D. Eckstein, G. Flucke, A. Geiser, I. Glushkov, P. Gunnellini, S. Habib, J. Hauk, G. Hellwig, H. Jung, M. Kasemann, P. Katsas, C. Kleinwort, H. Kluge, A. Knutsson, M. Krämer, D. Krücker, E. Kuznetsova, W. Lange, W. Lohmann, B. Lutz, R. Mankel, I. Marfin, M. Marienfeld, I.-A. Melzer-Pellmann, A. B. Meyer, J. Mnich, A. Mussgiller, S. Naumann-Emme, J. Olzem, H. Perrey, A. Petrukhin, D. Pitzl, A. Raspereza, P. M. Ribeiro Cipriano, C. Riedl, E. Ron, M. Rosin, J. Salfeld-Nebgen, R. Schmidt, T. Schoerner-Sadenius, N. Sen, A. Spiridonov, M. Stein, R. Walsh, C. Wissing, V. Blobel, J. Draeger, H. Enderle, J. Erfle, U. Gebbert, M. Görner, T. Hermanns, R. S. Höing, K. Kaschube, G. Kaussen, H. Kirschenmann, R. Klanner, J. Lange, B. Mura, F. Nowak, T. Peiffer, N. Pietsch, D. Rathjens, C. Sander, H. Schettler, P. Schleper, E. Schlieckau, A. Schmidt, M. Schröder, T. Schum, M. Seidel, V. Sola, H. Stadie, G. Steinbrück, J. Thomsen, L. Vanelderen, C. Barth, J. Berger, C. Böser, T. Chwalek, W. De Boer, A. Descroix, A. Dierlamm, M. Feindt, M. Guthoff, C. Hackstein, F. Hartmann, T. Hauth, M. Heinrich, H. Held, K. H. Hoffmann, S. Honc, I. Katkov, J. R. Komaragiri, P. Lobelle Pardo, D. Martschei, S. Mueller, Th. Müller, M. Niegel, A. Nürnberg, O. Oberst, A. Oehler, J. Ott, G. Quast, K. Rabbertz, F. Ratnikov, N. Ratnikova, S. Röcker, A. Scheurer, F.-P. Schilling, G. Schott, H. J. Simonis, F. M. Stober, D. Troendle, R. Ulrich, J. Wagner-Kuhr, S. Wayand, T. Weiler, M. Zeise, G. Daskalakis, T. Geralis, S. Kesisoglou, A. Kyriakis, D. Loukas, I. Manolakos, A. Markou, C. Markou, C. Mavrommatis, E. Ntomari, L. Gouskos, T. J. Mertzimekis, A. Panagiotou, N. Saoulidou, I. Evangelou, C. Foudas, P. Kokkas, N. Manthos, I. Papadopoulos, V. Patras, G. Bencze, C. Hajdu, P. Hidas, D. Horvath, F. Sikler, V. Veszpremi, G. Vesztergombi, N. Beni, S. Czellar, J. Molnar, J. Palinkas, Z. Szillasi, J. Karancsi, P. Raics, Z. L. Trocsanyi, B. Ujvari, S. B. Beri, V. Bhatnagar, N. Dhingra, R. Gupta, M. Kaur, M. Z. Mehta, N. Nishu, L. K. Saini, A. Sharma, J. B. Singh, Ashok Kumar, Arun Kumar, S. Ahuja, A. Bhardwaj, B. C. Choudhary, S. Malhotra, M. Naimuddin, K. Ranjan, V. Sharma, R. K. Shivpuri, S. Banerjee, S. Bhattacharya, S. Dutta, B. Gomber, Sa. Jain, Sh. Jain, R. Khurana, S. Sarkar, M. Sharan, A. Abdulsalam, R. K. Choudhury, D. Dutta, S. Kailas, V. Kumar, P. Mehta, A. K. Mohanty, L. M. Pant, P. Shukla, T. Aziz, S. Ganguly, M. Guchait, M. Maity, G. Majumder, K. Mazumdar, G. B. Mohanty, B. Parida, K. Sudhakar, N. Wickramage, S. Banerjee, S. Dugad, H. Arfaei, H. Bakhshiansohi, S. M. Etesami, A. Fahim, M. Hashemi, H. Hesari, A. Jafari, M. Khakzad, M. Mohammadi Najafabadi, S. Paktinat Mehdiabadi, B. Safarzadeh, M. Zeinali, M. Abbrescia, L. Barbone, C. Calabria, S. S. Chhibra, A. Colaleo, D. Creanza, N. De Filippis, M. De Palma, L. Fiore, G. Iaselli, L. Lusito, G. Maggi, M. Maggi, B. Marangelli, S. My, S. Nuzzo, N. Pacifico, A. Pompili, G. Pugliese, G. Selvaggi, L. Silvestris, G. Singh, R. Venditti, G. Zito, G. Abbiendi, A. C. Benvenuti, D. Bonacorsi, S. Braibant-Giacomelli, L. Brigliadori, P. Capiluppi, A. Castro, F. R. Cavallo, M. Cuffiani, G. M. Dallavalle, F. Fabbri, A. Fanfani, D. Fasanella, P. Giacomelli, C. Grandi, L. Guiducci, S. Marcellini, G. Masetti, M. Meneghelli, A. Montanari, F. L. Navarria, F. Odorici, A. Perrotta, F. Primavera, A. M. Rossi, T. Rovelli, G. P. Siroli, R. Travaglini, S. Albergo, G. Cappello, M. Chiorboli, S. Costa, R. Potenza, A. Tricomi, C. Tuve, G. Barbagli, V. Ciulli, C. Civinini, R. D’Alessandro, E. Focardi, S. Frosali, E. Gallo, S. Gonzi, M. Meschini, S. Paoletti, G. Sguazzoni, A. Tropiano, L. Benussi, S. Bianco, S. Colafranceschi, F. Fabbri, D. Piccolo, P. Fabbricatore, R. Musenich, S. Tosi, A. Benaglia, F. De Guio, L. Di Matteo, S. Fiorendi, S. Gennai, A. Ghezzi, S. Malvezzi, R. A. Manzoni, A. Martelli, A. Massironi, D. Menasce, L. Moroni, M. Paganoni, D. Pedrini, S. Ragazzi, N. Redaelli, S. Sala, T. Tabarelli de Fatis, S. Buontempo, C. A. Carrillo Montoya, N. Cavallo, A. De Cosa, O. Dogangun, F. Fabozzi, A. O. M. Iorio, L. Lista, S. Meola, M. Merola, P. Paolucci, P. Azzi, N. Bacchetta, D. Bisello, A. Branca, R. Carlin, P. Checchia, T. Dorigo, F. Gasparini, U. Gasparini, A. Gozzelino, K. Kanishchev, S. Lacaprara, I. Lazzizzera, M. Margoni, A. T. Meneguzzo, J. Pazzini, N. Pozzobon, P. Ronchese, F. Simonetto, E. Torassa, M. Tosi, S. Vanini, P. Zotto, A. Zucchetta, G. Zumerle, M. Gabusi, S. P. Ratti, C. Riccardi, P. Torre, P. Vitulo, M. Biasini, G. M. Bilei, L. Fanò, P. Lariccia, A. Lucaroni, G. Mantovani, M. Menichelli, A. Nappi, F. Romeo, A. Saha, A. Santocchia, A. Spiezia, S. Taroni, P. Azzurri, G. Bagliesi, T. Boccali, G. Broccolo, R. Castaldi, R. T. D’Agnolo, R. Dell’Orso, F. Fiori, L. Foà, A. Giassi, A. Kraan, F. Ligabue, T. Lomtadze, L. Martini, A. Messineo, F. Palla, A. Rizzi, A. T. Serban, P. Spagnolo, P. Squillacioti, R. Tenchini, G. Tonelli, A. Venturi, P. G. Verdini, L. Barone, F. Cavallari, D. Del Re, M. Diemoz, C. Fanelli, M. Grassi, E. Longo, P. Meridiani, F. Micheli, S. Nourbakhsh, G. Organtini, R. Paramatti, S. Rahatlou, M. Sigamani, L. Soffi, N. Amapane, R. Arcidiacono, S. Argiro, M. Arneodo, C. Biino, N. Cartiglia, M. Costa, N. Demaria, C. Mariotti, S. Maselli, E. Migliore, V. Monaco, M. Musich, M. M. Obertino, N. Pastrone, M. Pelliccioni, A. Potenza, A. Romero, R. Sacchi, A. Solano, A. Staiano, E. Usai, A. Vilela Pereira, S. Belforte, V. Candelise, F. Cossutti, G. Della Ricca, B. Gobbo, M. Marone, D. Montanino, A. Penzo, A. Schizzi, S. G. Heo, T. Y. Kim, S. K. Nam, S. Chang, D. H. Kim, G. N. Kim, D. J. Kong, H. Park, S. R. Ro, D. C. Son, T. Son, J. Y. Kim, Zero J. Kim, S. Song, S. Choi, D. Gyun, B. Hong, M. Jo, H. Kim, T. J. Kim, K. S. Lee, D. H. Moon, S. K. Park, M. Choi, J. H. Kim, C. Park, I. C. Park, S. Park, G. Ryu, Y. Cho, Y. Choi, Y. K. Choi, J. Goh, M. S. Kim, E. Kwon, B. Lee, J. Lee, S. Lee, H. Seo, I. Yu, M. J. Bilinskas, I. Grigelionis, M. Janulis, A. Juodagalvis, H. Castilla-Valdez, E. De La Cruz-Burelo, I. Heredia-de La Cruz, R. Lopez-Fernandez, R. Magaña Villalba, J. Martínez-Ortega, A. Sánchez-Hernández, L. M. Villasenor-Cendejas, S. Carrillo Moreno, F. Vazquez Valencia, H. A. Salazar Ibarguen, E. Casimiro Linares, A. Morelos Pineda, M. A. Reyes-Santos, D. Krofcheck, A. J. Bell, P. H. Butler, R. Doesburg, S. Reucroft, H. Silverwood, M. Ahmad, M. H. Ansari, M. I. Asghar, H. R. Hoorani, S. Khalid, W. A. Khan, T. Khurshid, S. Qazi, M. A. Shah, M. Shoaib, H. Bialkowska, B. Boimska, T. Frueboes, R. Gokieli, M. Górski, M. Kazana, K. Nawrocki, K. Romanowska-Rybinska, M. Szleper, G. Wrochna, P. Zalewski, G. Brona, K. Bunkowski, M. Cwiok, W. Dominik, K. Doroba, A. Kalinowski, M. Konecki, J. Krolikowski, N. Almeida, P. Bargassa, A. David, P. Faccioli, P. G. Ferreira Parracho, M. Gallinaro, J. Seixas, J. Varela, P. Vischia, I. Belotelov, P. Bunin, M. Gavrilenko, I. Golutvin, I. Gorbunov, A. Kamenev, V. Karjavin, G. Kozlov, A. Lanev, A. Malakhov, P. Moisenz, V. Palichik, V. Perelygin, S. Shmatov, V. Smirnov, A. Volodko, A. Zarubin, S. Evstyukhin, V. Golovtsov, Y. Ivanov, V. Kim, P. Levchenko, V. Murzin, V. Oreshkin, I. Smirnov, V. Sulimov, L. Uvarov, S. Vavilov, A. Vorobyev, An. Vorobyev, Yu. Andreev, A. Dermenev, S. Gninenko, N. Golubev, M. Kirsanov, N. Krasnikov, V. Matveev, A. Pashenkov, D. Tlisov, A. Toropin, V. Epshteyn, M. Erofeeva, V. Gavrilov, M. Kossov, N. Lychkovskaya, V. Popov, G. Safronov, S. Semenov, V. Stolin, E. Vlasov, A. Zhokin, A. Belyaev, E. Boos, M. Dubinin, L. Dudko, A. Ershov, A. Gribushin, V. Klyukhin, O. Kodolova, I. Lokhtin, A. Markina, S. Obraztsov, M. Perfilov, S. Petrushanko, A. Popov, L. Sarycheva, V. Savrin, A. Snigirev, V. Andreev, M. Azarkin, I. Dremin, M. Kirakosyan, A. Leonidov, G. Mesyats, S. V. Rusakov, A. Vinogradov, I. Azhgirey, I. Bayshev, S. Bitioukov, V. Grishin, V. Kachanov, D. Konstantinov, V. Krychkine, V. Petrov, R. Ryutin, A. Sobol, L. Tourtchanovitch, S. Troshin, N. Tyurin, A. Uzunian, A. Volkov, P. Adzic, M. Djordjevic, M. Ekmedzic, D. Krpic, J. Milosevic, M. Aguilar-Benitez, J. Alcaraz Maestre, P. Arce, C. Battilana, E. Calvo, M. Cerrada, M. Chamizo Llatas, N. Colino, B. De La Cruz, A. Delgado Peris, D. Domínguez Vázquez, C. Fernandez Bedoya, J. P. Fernández Ramos, A. Ferrando, J. Flix, M. C. Fouz, P. Garcia-Abia, O. Gonzalez Lopez, S. Goy Lopez, J. M. Hernandez, M. I. Josa, G. Merino, J. Puerta Pelayo, A. Quintario Olmeda, I. Redondo, L. Romero, J. Santaolalla, M. S. Soares, C. Willmott, C. Albajar, G. Codispoti, J. F. de Trocóniz, H. Brun, J. Cuevas, J. Fernandez Menendez, S. Folgueras, I. Gonzalez Caballero, L. Lloret Iglesias, J. Piedra Gomez, J. A. Brochero Cifuentes, I. J. Cabrillo, A. Calderon, S. H. Chuang, J. Duarte Campderros, M. Felcini, M. Fernandez, G. Gomez, J. Gonzalez Sanchez, A. Graziano, C. Jorda, A. Lopez Virto, J. Marco, R. Marco, C. Martinez Rivero, F. Matorras, F. J. Munoz Sanchez, T. Rodrigo, A. Y. Rodríguez-Marrero, A. Ruiz-Jimeno, L. Scodellaro, I. Vila, R. Vilar Cortabitarte, D. Abbaneo, E. Auffray, G. Auzinger, M. Bachtis, P. Baillon, A. H. Ball, D. Barney, J. F. Benitez, C. Bernet, G. Bianchi, P. Bloch, A. Bocci, A. Bonato, C. Botta, H. Breuker, T. Camporesi, G. Cerminara, T. Christiansen, J. A. Coarasa Perez, D. D’Enterria, A. Dabrowski, A. De Roeck, S. Di Guida, M. Dobson, N. Dupont-Sagorin, A. Elliott-Peisert, B. Frisch, W. Funk, G. Georgiou, M. Giffels, D. Gigi, K. Gill, D. Giordano, M. Giunta, F. Glege, R. Gomez-Reino Garrido, P. Govoni, S. Gowdy, R. Guida, M. Hansen, P. Harris, C. Hartl, J. Harvey, B. Hegner, A. Hinzmann, V. Innocente, P. Janot, K. Kaadze, E. Karavakis, K. Kousouris, P. Lecoq, Y.-J. Lee, P. Lenzi, C. Lourenço, N. Magini, T. Mäki, M. Malberti, L. Malgeri, M. Mannelli, L. Masetti, F. Meijers, S. Mersi, E. Meschi, R. Moser, M. U. Mozer, M. Mulders, P. Musella, E. Nesvold, T. Orimoto, L. Orsini, E. Palencia Cortezon, E. Perez, L. Perrozzi, A. Petrilli, A. Pfeiffer, M. Pierini, M. Pimiä, D. Piparo, G. Polese, L. Quertenmont, A. Racz, W. Reece, J. Rodrigues Antunes, G. Rolandi, C. Rovelli, M. Rovere, H. Sakulin, F. Santanastasio, C. Schäfer, C. Schwick, I. Segoni, S. Sekmen, A. Sharma, P. Siegrist, P. Silva, M. Simon, P. Sphicas, D. Spiga, A. Tsirou, G. I. Veres, J. R. Vlimant, H. K. Wöhri, S. D. Worm, W. D. Zeuner, W. Bertl, K. Deiters, W. Erdmann, K. Gabathuler, R. Horisberger, Q. Ingram, H. C. Kaestli, S. König, D. Kotlinski, U. Langenegger, F. Meier, D. Renker, T. Rohe, J. Sibille, L. Bäni, P. Bortignon, M. A. Buchmann, B. Casal, N. Chanon, A. Deisher, G. Dissertori, M. Dittmar, M. Donegà, M. Dünser, J. Eugster, K. Freudenreich, C. Grab, D. Hits, P. Lecomte, W. Lustermann, A. C. Marini, P. Martinez Ruiz del Arbol, N. Mohr, F. Moortgat, C. Nägeli, P. Nef, F. Nessi-Tedaldi, F. Pandolfi, L. Pape, F. Pauss, M. Peruzzi, F. J. Ronga, M. Rossini, L. Sala, A. K. Sanchez, A. Starodumov, B. Stieger, M. Takahashi, L. Tauscher, A. Thea, K. Theofilatos, D. Treille, C. Urscheler, R. Wallny, H. A. Weber, L. Wehrli, C. Amsler, V. Chiochia, S. De Visscher, C. Favaro, M. Ivova Rikova, B. Millan Mejias, P. Otiougova, P. Robmann, H. Snoek, S. Tupputi, M. Verzetti, Y. H. Chang, K. H. Chen, C. M. Kuo, S. W. Li, W. Lin, Z. K. Liu, Y. J. Lu, D. Mekterovic, A. P. Singh, R. Volpe, S. S. Yu, P. Bartalini, P. Chang, Y. H. Chang, Y. W. Chang, Y. Chao, K. F. Chen, C. Dietz, U. Grundler, W.-S. Hou, Y. Hsiung, K. Y. Kao, Y. J. Lei, R.-S. Lu, D. Majumder, E. Petrakou, X. Shi, J. G. Shiu, Y. M. Tzeng, X. Wan, M. Wang, A. Adiguzel, M. N. Bakirci, S. Cerci, C. Dozen, I. Dumanoglu, E. Eskut, S. Girgis, G. Gokbulut, E. Gurpinar, I. Hos, E. E. Kangal, T. Karaman, G. Karapinar, A. Kayis Topaksu, G. Onengut, K. Ozdemir, S. Ozturk, A. Polatoz, K. Sogut, D. Sunar Cerci, B. Tali, H. Topakli, L. N. Vergili, M. Vergili, I. V. Akin, T. Aliev, B. Bilin, S. Bilmis, M. Deniz, H. Gamsizkan, A. M. Guler, K. Ocalan, A. Ozpineci, M. Serin, R. Sever, U. E. Surat, M. Yalvac, E. Yildirim, M. Zeyrek, E. Gülmez, B. Isildak, M. Kaya, O. Kaya, S. Ozkorucuklu, N. Sonmez, K. Cankocak, L. Levchuk, F. Bostock, J. J. Brooke, E. Clement, D. Cussans, H. Flacher, R. Frazier, J. Goldstein, M. Grimes, G. P. Heath, H. F. Heath, L. Kreczko, S. Metson, D. M. Newbold, K. Nirunpong, A. Poll, S. Senkin, V. J. Smith, T. Williams, L. Basso, K. W. Bell, A. Belyaev, C. Brew, R. M. Brown, D. J. A. Cockerill, J. A. Coughlan, K. Harder, S. Harper, J. Jackson, B. W. Kennedy, E. Olaiya, D. Petyt, B. C. Radburn-Smith, C. H. Shepherd-Themistocleous, I. R. Tomalin, W. J. Womersley, R. Bainbridge, G. Ball, R. Beuselinck, O. Buchmuller, D. Colling, N. Cripps, M. Cutajar, P. Dauncey, G. Davies, M. Della Negra, W. Ferguson, J. Fulcher, D. Futyan, A. Gilbert, A. Guneratne Bryer, G. Hall, Z. Hatherell, J. Hays, G. Iles, M. Jarvis, G. Karapostoli, L. Lyons, A.-M. Magnan, J. Marrouche, B. Mathias, R. Nandi, J. Nash, A. Nikitenko, A. Papageorgiou, J. Pela, M. Pesaresi, K. Petridis, M. Pioppi, D. M. Raymond, S. Rogerson, A. Rose, M. J. Ryan, C. Seez, P. Sharp, A. Sparrow, M. Stoye, A. Tapper, M. Vazquez Acosta, T. Virdee, S. Wakefield, N. Wardle, T. Whyntie, M. Chadwick, J. E. Cole, P. R. Hobson, A. Khan, P. Kyberd, D. Leggat, D. Leslie, W. Martin, I. D. Reid, P. Symonds, L. Teodorescu, M. Turner, K. Hatakeyama, H. Liu, T. Scarborough, O. Charaf, C. Henderson, P. Rumerio, A. Avetisyan, T. Bose, C. Fantasia, A. Heister, J. St. John, P. Lawson, D. Lazic, J. Rohlf, D. Sperka, L. Sulak, J. Alimena, S. Bhattacharya, D. Cutts, A. Ferapontov, U. Heintz, S. Jabeen, G. Kukartsev, E. Laird, G. Landsberg, M. Luk, M. Narain, D. Nguyen, M. Segala, T. Sinthuprasith, T. Speer, K. V. Tsang, R. Breedon, G. Breto, M. Calderon De La Barca Sanchez, S. Chauhan, M. Chertok, J. Conway, R. Conway, P. T. Cox, J. Dolen, R. Erbacher, M. Gardner, R. Houtz, W. Ko, A. Kopecky, R. Lander, T. Miceli, D. Pellett, F. Ricci-tam, B. Rutherford, M. Searle, J. Smith, M. Squires, M. Tripathi, R. Vasquez Sierra, V. Andreev, D. Cline, R. Cousins, J. Duris, S. Erhan, P. Everaerts, C. Farrell, J. Hauser, M. Ignatenko, C. Jarvis, C. Plager, G. Rakness, P. Schlein, P. Traczyk, V. Valuev, M. Weber, J. Babb, R. Clare, M. E. Dinardo, J. Ellison, J. W. Gary, F. Giordano, G. Hanson, G. Y. Jeng, H. Liu, O. R. Long, A. Luthra, H. Nguyen, S. Paramesvaran, J. Sturdy, S. Sumowidagdo, R. Wilken, S. Wimpenny, W. Andrews, J. G. Branson, G. B. Cerati, S. Cittolin, D. Evans, F. Golf, A. Holzner, R. Kelley, M. Lebourgeois, J. Letts, I. Macneill, B. Mangano, S. Padhi, C. Palmer, G. Petrucciani, M. Pieri, M. Sani, V. Sharma, S. Simon, E. Sudano, M. Tadel, Y. Tu, A. Vartak, S. Wasserbaech, F. Würthwein, A. Yagil, J. Yoo, D. Barge, R. Bellan, C. Campagnari, M. D’Alfonso, T. Danielson, K. Flowers, P. Geffert, J. Incandela, C. Justus, P. Kalavase, S. A. Koay, D. Kovalskyi, V. Krutelyov, S. Lowette, N. Mccoll, V. Pavlunin, F. Rebassoo, J. Ribnik, J. Richman, R. Rossin, D. Stuart, W. To, C. West, A. Apresyan, A. Bornheim, Y. Chen, E. Di Marco, J. Duarte, M. Gataullin, Y. Ma, A. Mott, H. B. Newman, C. Rogan, M. Spiropulu, V. Timciuc, J. Veverka, R. Wilkinson, S. Xie, Y. Yang, R. Y. Zhu, B. Akgun, V. Azzolini, A. Calamba, R. Carroll, T. Ferguson, Y. Iiyama, D. W. Jang, Y. F. Liu, M. Paulini, H. Vogel, I. Vorobiev, J. P. Cumalat, B. R. Drell, C. J. Edelmaier, W. T. Ford, A. Gaz, B. Heyburn, E. Luiggi Lopez, J. G. Smith, K. Stenson, K. A. Ulmer, S. R. Wagner, J. Alexander, A. Chatterjee, N. Eggert, L. K. Gibbons, B. Heltsley, A. Khukhunaishvili, B. Kreis, N. Mirman, G. Nicolas Kaufman, J. R. Patterson, A. Ryd, E. Salvati, W. Sun, W. D. Teo, J. Thom, J. Thompson, J. Tucker, J. Vaughan, Y. Weng, L. Winstrom, P. Wittich, D. Winn, S. Abdullin, M. Albrow, J. Anderson, L. A. T. Bauerdick, A. Beretvas, J. Berryhill, P. C. Bhat, I. Bloch, K. Burkett, J. N. Butler, V. Chetluru, H. W. K. Cheung, F. Chlebana, V. D. Elvira, I. Fisk, J. Freeman, Y. Gao, D. Green, O. Gutsche, J. Hanlon, R. M. Harris, J. Hirschauer, B. Hooberman, S. Jindariani, M. Johnson, U. Joshi, B. Kilminster, B. Klima, S. Kunori, S. Kwan, C. Leonidopoulos, J. Linacre, D. Lincoln, R. Lipton, J. Lykken, K. Maeshima, J. M. Marraffino, S. Maruyama, D. Mason, P. McBride, K. Mishra, S. Mrenna, Y. Musienko, C. Newman-Holmes, V. O’Dell, O. Prokofyev, E. Sexton-Kennedy, S. Sharma, W. J. Spalding, L. Spiegel, P. Tan, L. Taylor, S. Tkaczyk, N. V. Tran, L. Uplegger, E. W. Vaandering, R. Vidal, J. Whitmore, W. Wu, F. Yang, F. Yumiceva, J. C. Yun, D. Acosta, P. Avery, D. Bourilkov, M. Chen, T. Cheng, S. Das, M. De Gruttola, G. P. Di Giovanni, D. Dobur, A. Drozdetskiy, R. D. Field, M. Fisher, Y. Fu, I. K. Furic, J. Gartner, J. Hugon, B. Kim, J. Konigsberg, A. Korytov, A. Kropivnitskaya, T. Kypreos, J. F. Low, K. Matchev, P. Milenovic, G. Mitselmakher, L. Muniz, R. Remington, A. Rinkevicius, P. Sellers, N. Skhirtladze, M. Snowball, J. Yelton, M. Zakaria, V. Gaultney, S. Hewamanage, L. M. Lebolo, S. Linn, P. Markowitz, G. Martinez, J. L. Rodriguez, T. Adams, A. Askew, J. Bochenek, J. Chen, B. Diamond, S. V. Gleyzer, J. Haas, S. Hagopian, V. Hagopian, M. Jenkins, K. F. Johnson, H. Prosper, V. Veeraraghavan, M. Weinberg, M. M. Baarmand, B. Dorney, M. Hohlmann, H. Kalakhety, I. Vodopiyanov, M. R. Adams, I. M. Anghel, L. Apanasevich, Y. Bai, V. E. Bazterra, R. R. Betts, I. Bucinskaite, J. Callner, R. Cavanaugh, O. Evdokimov, L. Gauthier, C. E. Gerber, D. J. Hofman, S. Khalatyan, F. Lacroix, M. Malek, C. O’Brien, C. Silkworth, D. Strom, P. Turner, N. Varelas, U. Akgun, E. A. Albayrak, B. Bilki, W. Clarida, F. Duru, S. Griffiths, J.-P. Merlo, H. Mermerkaya, A. Mestvirishvili, A. Moeller, J. Nachtman, C. R. Newsom, E. Norbeck, Y. Onel, F. Ozok, S. Sen, E. Tiras, J. Wetzel, T. Yetkin, K. Yi, B. A. Barnett, B. Blumenfeld, S. Bolognesi, D. Fehling, G. Giurgiu, A. V. Gritsan, Z. J. Guo, G. Hu, P. Maksimovic, S. Rappoccio, M. Swartz, A. Whitbeck, P. Baringer, A. Bean, G. Benelli, O. Grachov, R. P. Kenny Iii, M. Murray, D. Noonan, S. Sanders, R. Stringer, G. Tinti, J. S. Wood, V. Zhukova, A. F. Barfuss, T. Bolton, I. Chakaberia, A. Ivanov, S. Khalil, M. Makouski, Y. Maravin, S. Shrestha, I. Svintradze, J. Gronberg, D. Lange, D. Wright, A. Baden, M. Boutemeur, B. Calvert, S. C. Eno, J. A. Gomez, N. J. Hadley, R. G. Kellogg, M. Kirn, T. Kolberg, Y. Lu, M. Marionneau, A. C. Mignerey, K. Pedro, A. Peterman, A. Skuja, J. Temple, M. B. Tonjes, S. C. Tonwar, E. Twedt, A. Apyan, G. Bauer, J. Bendavid, W. Busza, E. Butz, I. A. Cali, M. Chan, V. Dutta, G. Gomez Ceballos, M. Goncharov, K. A. Hahn, Y. Kim, M. Klute, K. Krajczar, W. Li, P. D. Luckey, T. Ma, S. Nahn, C. Paus, D. Ralph, C. Roland, G. Roland, M. Rudolph, G. S. F. Stephans, F. Stöckli, K. Sumorok, K. Sung, D. Velicanu, E. A. Wenger, R. Wolf, B. Wyslouch, M. Yang, Y. Yilmaz, A. S. Yoon, M. Zanetti, S. I. Cooper, B. Dahmes, A. De Benedetti, G. Franzoni, A. Gude, S. C. Kao, K. Klapoetke, Y. Kubota, J. Mans, N. Pastika, R. Rusack, M. Sasseville, A. Singovsky, N. Tambe, J. Turkewitz, L. M. Cremaldi, R. Kroeger, L. Perera, R. Rahmat, D. A. Sanders, E. Avdeeva, K. Bloom, S. Bose, J. Butt, D. R. Claes, A. Dominguez, M. Eads, J. Keller, I. Kravchenko, J. Lazo-Flores, H. Malbouisson, S. Malik, G. R. Snow, U. Baur, A. Godshalk, I. Iashvili, S. Jain, A. Kharchilava, A. Kumar, S. P. Shipkowski, K. Smith, G. Alverson, E. Barberis, D. Baumgartel, M. Chasco, J. Haley, D. Nash, D. Trocino, D. Wood, J. Zhang, A. Anastassov, A. Kubik, N. Mucia, N. Odell, R. A. Ofierzynski, B. Pollack, A. Pozdnyakov, M. Schmitt, S. Stoynev, M. Velasco, S. Won, L. Antonelli, D. Berry, A. Brinkerhoff, M. Hildreth, C. Jessop, D. J. Karmgard, J. Kolb, K. Lannon, W. Luo, S. Lynch, N. Marinelli, D. M. Morse, T. Pearson, M. Planer, R. Ruchti, J. Slaunwhite, N. Valls, M. Wayne, M. Wolf, B. Bylsma, L. S. Durkin, C. Hill, R. Hughes, K. Kotov, T. Y. Ling, D. Puigh, M. Rodenburg, C. Vuosalo, G. Williams, B. L. Winer, N. Adam, E. Berry, P. Elmer, D. Gerbaudo, V. Halyo, P. Hebda, J. Hegeman, A. Hunt, P. Jindal, D. Lopes Pegna, P. Lujan, D. Marlow, T. Medvedeva, M. Mooney, J. Olsen, P. Piroué, X. Quan, A. Raval, B. Safdi, H. Saka, D. Stickland, C. Tully, J. S. Werner, A. Zuranski, J. G. Acosta, E. Brownson, X. T. Huang, A. Lopez, H. Mendez, S. Oliveros, J. E. Ramirez Vargas, A. Zatserklyaniy, E. Alagoz, V. E. Barnes, D. Benedetti, G. Bolla, D. Bortoletto, M. De Mattia, A. Everett, Z. Hu, M. Jones, O. Koybasi, M. Kress, A. T. Laasanen, N. Leonardo, V. Maroussov, P. Merkel, D. H. Miller, N. Neumeister, I. Shipsey, D. Silvers, A. Svyatkovskiy, M. Vidal Marono, H. D. Yoo, J. Zablocki, Y. Zheng, S. Guragain, N. Parashar, A. Adair, C. Boulahouache, K. M. Ecklund, F. J. M. Geurts, B. P. Padley, R. Redjimi, J. Roberts, J. Zabel, B. Betchart, A. Bodek, Y. S. Chung, R. Covarelli, P. de Barbaro, R. Demina, Y. Eshaq, T. Ferbel, A. Garcia-Bellido, P. Goldenzweig, J. Han, A. Harel, D. C. Miner, D. Vishnevskiy, M. Zielinski, A. Bhatti, R. Ciesielski, L. Demortier, K. Goulianos, G. Lungu, S. Malik, C. Mesropian, S. Arora, A. Barker, J. P. Chou, C. Contreras-Campana, E. Contreras-Campana, D. Duggan, D. Ferencek, Y. Gershtein, R. Gray, E. Halkiadakis, D. Hidas, A. Lath, S. Panwalkar, M. Park, R. Patel, V. Rekovic, J. Robles, K. Rose, S. Salur, S. Schnetzer, C. Seitz, S. Somalwar, R. Stone, S. Thomas, G. Cerizza, M. Hollingsworth, S. Spanier, Z. C. Yang, A. York, R. Eusebi, W. Flanagan, J. Gilmore, T. Kamon, V. Khotilovich, R. Montalvo, I. Osipenkov, Y. Pakhotin, A. Perloff, J. Roe, A. Safonov, T. Sakuma, S. Sengupta, I. Suarez, A. Tatarinov, D. Toback, N. Akchurin, J. Damgov, C. Dragoiu, P. R. Dudero, C. Jeong, K. Kovitanggoon, S. W. Lee, T. Libeiro, Y. Roh, I. Volobouev, E. Appelt, A. G. Delannoy, C. Florez, S. Greene, A. Gurrola, W. Johns, C. Johnston, P. Kurt, C. Maguire, A. Melo, M. Sharma, P. Sheldon, B. Snook, S. Tuo, J. Velkovska, M. W. Arenton, M. Balazs, S. Boutle, B. Cox, B. Francis, J. Goodell, R. Hirosky, A. Ledovskoy, C. Lin, C. Neu, J. Wood, R. Yohay, S. Gollapinni, R. Harr, P. E. Karchin, C. Kottachchi Kankanamge Don, P. Lamichhane, A. Sakharov, M. Anderson, D. Belknap, L. Borrello, D. Carlsmith, M. Cepeda, S. Dasu, E. Friis, L. Gray, K. S. Grogg, M. Grothe, R. Hall-Wilton, M. Herndon, A. Hervé, P. Klabbers, J. Klukas, A. Lanaro, C. Lazaridis, J. Leonard, R. Loveless, A. Mohapatra, I. Ojalvo, F. Palmonari, G. A. Pierro, I. Ross, A. Savin, W. H. Smith, J. Swanson

**Affiliations:** 1 CERN, Geneva, Switzerland; 2 Yerevan Physics Institute, Yerevan, Armenia; 3 Institut für Hochenergiephysik der OeAW, Wien, Austria; 4 National Centre for Particle and High Energy Physics, Minsk, Belarus; 5 Universiteit Antwerpen, Antwerpen, Belgium; 6 Vrije Universiteit Brussel, Brussel, Belgium; 7 Université Libre de Bruxelles, Bruxelles, Belgium; 8 Ghent University, Ghent, Belgium; 9 Université Catholique de Louvain, Louvain-la-Neuve, Belgium; 10 Université de Mons, Mons, Belgium; 11 Centro Brasileiro de Pesquisas Fisicas, Rio de Janeiro, Brazil; 12 Universidade do Estado do Rio de Janeiro, Rio de Janeiro, Brazil; 13 Instituto de Fisica Teorica, Universidade Estadual Paulista, Sao Paulo, Brazil; 14 Institute for Nuclear Research and Nuclear Energy, Sofia, Bulgaria; 15 University of Sofia, Sofia, Bulgaria; 16 Institute of High Energy Physics, Beijing, China; 17 State Key Lab. of Nucl. Phys. and Tech., Peking University, Beijing, China; 18 Universidad de Los Andes, Bogota, Colombia; 19 Technical University of Split, Split, Croatia; 20 University of Split, Split, Croatia; 21 Institute Rudjer Boskovic, Zagreb, Croatia; 22 University of Cyprus, Nicosia, Cyprus; 23 Charles University, Prague, Czech Republic; 24 Academy of Scientific Research and Technology of the Arab Republic of Egypt, Egyptian Network of High Energy Physics, Cairo, Egypt; 25 National Institute of Chemical Physics and Biophysics, Tallinn, Estonia; 26 Department of Physics, University of Helsinki, Helsinki, Finland; 27 Helsinki Institute of Physics, Helsinki, Finland; 28 Lappeenranta University of Technology, Lappeenranta, Finland; 29 DSM/IRFU, CEA/Saclay, Gif-sur-Yvette, France; 30 Laboratoire Leprince-Ringuet, Ecole Polytechnique, IN2P3-CNRS, Palaiseau, France; 31 Institut Pluridisciplinaire Hubert Curien, Université de Strasbourg, Université de Haute Alsace Mulhouse, CNRS/IN2P3, Strasbourg, France; 32 Centre de Calcul de l’Institut National de Physique Nucleaire et de Physique des Particules, CNRS/IN2P3, Villeurbanne, France; 33 Université de Lyon, Université Claude Bernard Lyon 1, CNRS-IN2P3, Institut de Physique Nucléaire de Lyon, Villeurbanne, France; 34 Institute of High Energy Physics and Informatization, Tbilisi State University, Tbilisi, Georgia; 35 I. Physikalisches Institut, RWTH Aachen University, Aachen, Germany; 36 III. Physikalisches Institut A, RWTH Aachen University, Aachen, Germany; 37 III. Physikalisches Institut B, RWTH Aachen University, Aachen, Germany; 38 Deutsches Elektronen-Synchrotron, Hamburg, Germany; 39 University of Hamburg, Hamburg, Germany; 40 Institut für Experimentelle Kernphysik, Karlsruhe, Germany; 41 Institute of Nuclear Physics “Demokritos”, Aghia Paraskevi, Greece; 42 University of Athens, Athens, Greece; 43 University of Ioánnina, Ioánnina, Greece; 44 KFKI Research Institute for Particle and Nuclear Physics, Budapest, Hungary; 45 Institute of Nuclear Research ATOMKI, Debrecen, Hungary; 46 University of Debrecen, Debrecen, Hungary; 47 Panjab University, Chandigarh, India; 48 University of Delhi, Delhi, India; 49 Saha Institute of Nuclear Physics, Kolkata, India; 50 Bhabha Atomic Research Centre, Mumbai, India; 51 Tata Institute of Fundamental Research - EHEP, Mumbai, India; 52 Tata Institute of Fundamental Research - HECR, Mumbai, India; 53 Institute for Research in Fundamental Sciences (IPM), Tehran, Iran; 54 INFN Sezione di Bari, Bari, Italy; 55 Università di Bari, Bari, Italy; 56 Politecnico di Bari, Bari, Italy; 57 INFN Sezione di Bologna, Bologna, Italy; 58 Università di Bologna, Bologna, Italy; 59 INFN Sezione di Catania, Catania, Italy; 60 Università di Catania, Catania, Italy; 61 INFN Sezione di Firenze, Firenze, Italy; 62 Università di Firenze, Firenze, Italy; 63 INFN Laboratori Nazionali di Frascati, Frascati, Italy; 64 INFN Sezione di Genova, Genova, Italy; 65 Università di Genova, Genova, Italy; 66 INFN Sezione di Milano-Bicocca, Milano, Italy; 67 Università di Milano-Bicocca, Milano, Italy; 68 INFN Sezione di Napoli, Napoli, Italy; 69 Università di Napoli “Federico II”, Napoli, Italy; 70 INFN Sezione di Padova, Padova, Italy; 71 Università di Padova, Padova, Italy; 72 Università di Trento (Trento), Padova, Italy; 73 INFN Sezione di Pavia, Pavia, Italy; 74 Università di Pavia, Pavia, Italy; 75 INFN Sezione di Perugia, Perugia, Italy; 76 Università di Perugia, Perugia, Italy; 77 INFN Sezione di Pisa, Pisa, Italy; 78 Università di Pisa, Pisa, Italy; 79 Scuola Normale Superiore di Pisa, Pisa, Italy; 80 INFN Sezione di Roma, Roma, Italy; 81 Università di Roma, Roma, Italy; 82 INFN Sezione di Torino, Torino, Italy; 83 Università di Torino, Torino, Italy; 84 Università del Piemonte Orientale (Novara), Torino, Italy; 85 INFN Sezione di Trieste, Trieste, Italy; 86 Università di Trieste, Trieste, Italy; 87 Kangwon National University, Chunchon, Korea; 88 Kyungpook National University, Daegu, Korea; 89 Institute for Universe and Elementary Particles, Chonnam National University, Kwangju, Korea; 90 Korea University, Seoul, Korea; 91 University of Seoul, Seoul, Korea; 92 Sungkyunkwan University, Suwon, Korea; 93 Vilnius University, Vilnius, Lithuania; 94 Centro de Investigacion y de Estudios Avanzados del IPN, Mexico City, Mexico; 95 Universidad Iberoamericana, Mexico City, Mexico; 96 Benemerita Universidad Autonoma de Puebla, Puebla, Mexico; 97 Universidad Autónoma de San Luis Potosí, San Luis Potosí, Mexico; 98 University of Auckland, Auckland, New Zealand; 99 University of Canterbury, Christchurch, New Zealand; 100 National Centre for Physics, Quaid-I-Azam University, Islamabad, Pakistan; 101 National Centre for Nuclear Research, Swierk, Poland; 102 Institute of Experimental Physics, Faculty of Physics, University of Warsaw, Warsaw, Poland; 103 Laboratório de Instrumentação e Física Experimental de Partículas, Lisboa, Portugal; 104 Joint Institute for Nuclear Research, Dubna, Russia; 105 Petersburg Nuclear Physics Institute, Gatchina (St. Petersburg), Russia; 106 Institute for Nuclear Research, Moscow, Russia; 107 Institute for Theoretical and Experimental Physics, Moscow, Russia; 108 Moscow State University, Moscow, Russia; 109 P.N. Lebedev Physical Institute, Moscow, Russia; 110 State Research Center of Russian Federation, Institute for High Energy Physics, Protvino, Russia; 111 Faculty of Physics and Vinca Institute of Nuclear Sciences, University of Belgrade, Belgrade, Serbia; 112 Centro de Investigaciones Energéticas Medioambientales y Tecnológicas (CIEMAT), Madrid, Spain; 113 Universidad Autónoma de Madrid, Madrid, Spain; 114 Universidad de Oviedo, Oviedo, Spain; 115 Instituto de Física de Cantabria (IFCA), CSIC-Universidad de Cantabria, Santander, Spain; 116 CERN, European Organization for Nuclear Research, Geneva, Switzerland; 117 Paul Scherrer Institut, Villigen, Switzerland; 118 Institute for Particle Physics, ETH Zurich, Zurich, Switzerland; 119 Universität Zürich, Zurich, Switzerland; 120 National Central University, Chung-Li, Taiwan; 121 National Taiwan University (NTU), Taipei, Taiwan; 122 Cukurova University, Adana, Turkey; 123 Middle East Technical University, Physics Department, Ankara, Turkey; 124 Bogazici University, Istanbul, Turkey; 125 Istanbul Technical University, Istanbul, Turkey; 126 National Scientific Center, Kharkov Institute of Physics and Technology, Kharkov, Ukraine; 127 University of Bristol, Bristol, United Kingdom; 128 Rutherford Appleton Laboratory, Didcot, United Kingdom; 129 Imperial College, London, United Kingdom; 130 Brunel University, Uxbridge, United Kingdom; 131 Baylor University, Waco, USA; 132 The University of Alabama, Tuscaloosa, USA; 133 Boston University, Boston, USA; 134 Brown University, Providence, USA; 135 University of California, Davis, Davis, USA; 136 University of California, Los Angeles, Los Angeles, USA; 137 University of California, Riverside, Riverside, USA; 138 University of California, San Diego, La Jolla, USA; 139 University of California, Santa Barbara, Santa Barbara, USA; 140 California Institute of Technology, Pasadena, USA; 141 Carnegie Mellon University, Pittsburgh, USA; 142 University of Colorado at Boulder, Boulder, USA; 143 Cornell University, Ithaca, USA; 144 Fairfield University, Fairfield, USA; 145 Fermi National Accelerator Laboratory, Batavia, USA; 146 University of Florida, Gainesville, USA; 147 Florida International University, Miami, USA; 148 Florida State University, Tallahassee, USA; 149 Florida Institute of Technology, Melbourne, USA; 150 University of Illinois at Chicago (UIC), Chicago, USA; 151 The University of Iowa, Iowa City, USA; 152 Johns Hopkins University, Baltimore, USA; 153 The University of Kansas, Lawrence, USA; 154 Kansas State University, Manhattan, USA; 155 Lawrence Livermore National Laboratory, Livermore, USA; 156 University of Maryland, College Park, USA; 157 Massachusetts Institute of Technology, Cambridge, USA; 158 University of Minnesota, Minneapolis, USA; 159 University of Mississippi, Oxford, USA; 160 University of Nebraska-Lincoln, Lincoln, USA; 161 State University of New York at Buffalo, Buffalo, USA; 162 Northeastern University, Boston, USA; 163 Northwestern University, Evanston, USA; 164 University of Notre Dame, Notre Dame, USA; 165 The Ohio State University, Columbus, USA; 166 Princeton University, Princeton, USA; 167 University of Puerto Rico, Mayaguez, USA; 168 Purdue University, West Lafayette, USA; 169 Purdue University Calumet, Hammond, USA; 170 Rice University, Houston, USA; 171 University of Rochester, Rochester, USA; 172 The Rockefeller University, New York, USA; 173 Rutgers the State University of New Jersey, Piscataway, USA; 174 University of Tennessee, Knoxville, USA; 175 Texas A&M University, College Station, USA; 176 Texas Tech University, Lubbock, USA; 177 Vanderbilt University, Nashville, USA; 178 University of Virginia, Charlottesville, USA; 179 Wayne State University, Detroit, USA; 180 University of Wisconsin, Madison, USA

## Abstract

A measurement is presented of the relative prompt production rate of *χ*_c2_ and *χ*_c1_ with 4.6 fb^−1^ of data collected by the CMS experiment at the LHC in pp collisions at $\sqrt{s}= 7~\mathrm{TeV}$. The two states are measured via their radiative decays *χ*_c_→J/*ψ*+*γ*, with the photon converting into an e^+^e^−^ pair for J/*ψ* rapidity |*y*(J/*ψ*)|<1.0 and photon transverse momentum *p*_T_(*γ*)>0.5 GeV/*c*. The measurement is given for six intervals of *p*_T_(J/*ψ*) between 7 and 25 GeV/*c*. The results are compared to theoretical predictions.

## Introduction

Understanding charmonium production in hadronic collisions is a challenge for quantum chromodynamics (QCD). The J/*ψ* production cross section measurements at the Tevatron [1, 2] were found to disagree by about a factor of 50 with theoretical color-singlet calculations [3]. Soon after, the CDF experiment reported a *χ*_c2_/*χ*_c1_ cross section ratio that extended up to *p*_T_(J/*ψ*)≃10 GeV/*c*, where *p*_T_ is the transverse momentum, and favored *χ*_c1_ production over *χ*_c2_ [4]. The cross section ratio was also studied recently at the Large Hadron Collider (LHC) in Ref. [5]. These measurements independently suggest that charmonium production cannot be explained through relatively simple models.

This paper presents a measurement of the prompt *χ*_c2_/*χ*_c1_ cross section ratio by the Compact Muon Solenoid (CMS) experiment at the LHC in pp collisions at a center-of-mass energy of 7 TeV. Prompt refers to the production of *χ*_c_ mesons that originate from the primary pp interaction point, as opposed to the ones from the decay of B hadrons. Prompt production includes both directly produced *χ*_c_ and also indirectly produced *χ*_c_ from the decays of short-lived intermediate states, e.g. the radiative decay of the *ψ*(2*S*). The measurement is based on the reconstruction of the *χ*_c_ radiative decays to J/*ψ*+*γ*, with the low transverse momentum photons (less than 5 GeV/*c*) being detected through their conversion into electron–positron pairs. The analysis uses data collected in 2011, corresponding to a total integrated luminosity of 4.6 fb^−1^. When estimating acceptance and efficiencies, we assume that the *χ*_c2_ and *χ*_c1_ are produced unpolarized, and we supply the correction factors needed to modify the results for several different polarization scenarios.

Due to the extended reach in transverse momentum made possible by the LHC energies, the cross section ratio measurement is expected to discriminate between different predictions, such as those provided by the *k*_T_-factorization [6] and next-to-leading order nonrelativistic QCD (NRQCD) [7] theoretical approaches.

The strength of the ratio measurement is that most theoretical uncertainties cancel, including the quark masses, the value of the strong coupling constant *α*_*s*_, as well as experimental uncertainties on quantities such as integrated luminosity, trigger efficiencies, and, in part, reconstruction efficiency. Therefore, this ratio can be regarded as an important reference measurement to test the validity of various theoretical quarkonium production models. With this paper, we hope to provide further guidance for future calculations.

## CMS detector

A detailed description of the CMS apparatus is given in Ref. [8]. Here we provide a short summary of the detectors relevant for this measurement.

The central feature of the CMS apparatus is a superconducting solenoid of 6 m internal diameter. Within the field volume are the silicon pixel and strip tracker, the crystal electromagnetic calorimeter and the brass/scintillator hadron calorimeter. Muons are measured in gas-ionization detectors embedded in the steel return yoke. In addition to the barrel and endcap detectors, CMS has extensive forward calorimetry.

The inner tracker measures charged particles within the pseudorapidity range |*η*|<2.5, where *η*=−ln[tan(*θ*/2)], and *θ* is the polar angle measured from the beam axis. It consists of 1440 silicon pixel and 15 148 silicon strip detector modules. In the central region, modules are arranged in 13 measurement layers. It provides an impact parameter resolution of ∼15 μm.

Muons are measured in the pseudorapidity range |*η*|<2.4, with detection planes made using three technologies: drift tubes, cathode strip chambers, and resistive plate chambers. Matching the muons to the tracks measured in the silicon tracker results in a transverse momentum resolution between 1 and 1.5 %, for *p*_T_ values up to 50 GeV/*c*.

The first level (L1) of the CMS trigger system, composed of custom hardware processors, uses information from the calorimeters and muon detectors to select the most interesting events. The high-level trigger (HLT) processor farm further decreases the event rate from around 100 kHz to around 300 Hz, before data storage. The rate of HLT triggers relevant for this analysis was in the range 5–10 Hz. We analyzed about 60 million such triggers.

## Experimental method

We select *χ*_c1_ and *χ*_c2_ candidates by searching for their radiative decays into the J/*ψ*+*γ* final state, with the J/*ψ* decaying into two muons. The *χ*_c0_ has too small a branching fraction into this final state to perform a useful measurement, but we consider it in the modeling of the signal lineshape. Given the small difference between the J/*ψ* mass, 3096.916±0.011 MeV/*c* ^2^, and the *χ*_c1_ and *χ*_c2_ masses, 3510.66±0.07 MeV/*c* ^2^ and 3556.20±0.09 MeV/*c* ^2^, respectively [9], the detector must be able to reconstruct photons of low transverse momentum. In addition, excellent photon momentum resolution is needed to resolve the two states. In the center-of-mass frame of the *χ*_c_ states, the photon has an energy of 390 MeVwhen emitted by a *χ*_c1_ and 430 MeV when emitted by a *χ*_c2_. This results in most of the photons having a *p*_T_ in the laboratory frame smaller than 6 GeV/*c*. The precision of the cross section ratio measurement depends crucially on the experimental photon energy resolution, which must be good enough to separate the two states. A very accurate measurement of the photon energy is obtained by measuring electron–positron pairs originating from a photon conversion in the beampipe or the inner layers of the silicon tracker. The superior resolution of this approach, compared to a calorimetric energy measurement, comes at the cost of a reduced yield due to the small probability for a conversion to occur in the innermost part of the tracker detector and, more importantly, by the small reconstruction efficiency for low transverse momentum tracks whose origin is displaced with respect to the beam axis. Nevertheless, because of the high *χ*_c_ production cross section at the LHC, the use of conversions leads to the most precise result.

For each *χ*_c1,2_ candidate, we evaluate the mass difference Δ*m*=*m*_*μμγ*_−*m*_*μμ*_ between the dimuon-plus-photon invariant mass, *m*_*μμγ*_, and the dimuon invariant mass, *m*_*μμ*_. We use the quantity *Q*=Δ*m*+*m*_J/*ψ*_, where *m*_J/*ψ*_ is the world-average mass of the J/*ψ* from Ref. [9], as a convenient variable for plotting the invariant-mass distribution. We perform an unbinned maximum-likelihood fit to the *Q* spectrum to extract the yield of prompt *χ*_c1_ and *χ*_c2_ as a function of the transverse momentum of the J/*ψ*. A correction is applied for the differing acceptances for the two states. Our results are given in terms of the prompt production ratio *R*_p_, defined as

$$R_\mathrm{p} \equiv \frac{\sigma(\mathrm{p}\mathrm{p}\to\chi_{\mathrm{c}2}+X ) \mathcal{B}(\chi_{\mathrm{c}2}\to{\mathrm{J}/\psi}+ \gamma) }{ \sigma(\mathrm{p}\mathrm{p}\to\chi_{\mathrm{c}1}+X ) \mathcal {B}(\chi_{\mathrm{c}1}\to{\mathrm{J}/\psi}+ \gamma) } =\frac{N_{\chi_{\mathrm{c}2}}}{N_{\chi_{\mathrm{c}1}}} \cdot\frac {\varepsilon_1}{\varepsilon_2} , $$

where *σ*(pp→*χ*_c_+*X*) are the *χ*_c_ production cross sections, $\mathcal{B}(\chi _{\mathrm{c}}\to{\mathrm{J}/\psi}+ \gamma)$ are the *χ*_c_ branching fractions, $N_{\chi_{\mathrm{c}i}}$ are the number of candidates of each type obtained from the fit, and *ε*_1_/*ε*_2_ is the ratio of the efficiencies for the two *χ*_c_ states. The branching fractions $\mathcal{B}(\chi_{\mathrm{c}1,2} \to{\mathrm{J}/\psi}+ \gamma)$, taken from Ref. [9], are also used to calculate the ratio of production cross sections.

## Event reconstruction and selection

In order to select *χ*_c_ signal events, a dimuon trigger is used to record events containing the decay J/*ψ*→*μμ*. The L1 selection requires two muons without an explicit constraint on their transverse momentum. At the HLT, opposite-charge dimuons are reconstructed and the dimuon rapidity *y*(*μμ*) is required to satisfy |*y*(*μμ*)|<1.0, while the dimuon *p*_T_ must exceed a threshold that increased from 6.5 to 10 GeV/*c* as the trigger configuration evolved to cope with the instantaneous luminosity increase. Events containing dimuon candidates with invariant mass from 2.95 to 3.25 GeV/*c* ^2^ are recorded. Our data sample consists of events where multiple pp interactions occur. At each bunch crossing, an average of six primary vertices is reconstructed, one of them related to the interaction that produces the *χ*_c_ in the final state, the others related to softer collisions (pileup).

In the J/*ψ* selection, the muon tracks are required to pass the following criteria. They must have at least 11 hits in the tracker, with at least two in the pixel layers, to remove background from decays-in-flight. The *χ* ^2^ per degree of freedom of the track fit must be less than 1.8. To remove background from cosmic-ray muons, the tracks must intersect a cylindrical volume of radius 4 cm and total length 70 cm, centered at the nominal interaction point and with its axis parallel to the beam line. Muon candidate tracks are required to have *p*_T_>3.3 GeV/*c*, |*η*|≤1.3 and match a well-reconstructed segment in at least one muon detector [10]. Muons with opposite charges are paired. The two muon trajectories are fitted with a common vertex constraint, and events are retained if the fit *χ* ^2^ probability is larger than 1 %. If more than one muon pair is found in an event, only the pair with the largest vertex *χ* ^2^ probability is selected. For the final *χ*_c1_ and *χ*_c2_ selection, a dimuon candidate must have an invariant mass between 3.0 and 3.2 GeV/*c* ^2^ and |*y*|<1.0.

In order to restrict the measurement to the prompt J/*ψ* signal component, the pseudo-proper decay length of the J/*ψ*(*ℓ*_J/*ψ*_), defined as *ℓ*_J/*ψ*_=*L*_*xy*_⋅*m*_J/*ψ*_/*p*_T_(J/*ψ*), where *L*_*xy*_ is the most probable transverse decay length in the laboratory frame [11], is required to be less than 30 μm. In the region *ℓ*_J/*ψ*_<30 μm, we estimate, from the observed *ℓ*_J/*ψ*_ distribution, a contamination of the nonprompt component (originating from the decays of B hadrons) of about 0.7 %, which has a negligible impact on the total systematic uncertainty.

To reconstruct the photon from radiative decays, we use the tracker-based conversion reconstruction described in Refs. [12–14]. We summarize the method here, mentioning the further requirements needed to specialize the conversion reconstruction algorithm to the *χ*_c_ case. The algorithm relies on the capability of iterative tracking to efficiently reconstruct displaced and low transverse momentum tracks. Photon conversions are characterized by an electron–positron pair originating from a common vertex. The *e* ^+^ *e* ^−^ invariant mass must be consistent with zero within its uncertainties and the two tracks are required to be parallel at the conversion point.

Opposite-sign track pairs are first required to have more than four hits and a normalized *χ* ^2^ less than 10. Then the reconstruction algorithm exploits the conversion-pair signature to distinguish between genuine and misidentified background pairs. Information from the calorimeters is not used for conversion reconstruction in our analysis. The primary pp collision vertex associated with the photon conversion, see below, is required to lie outside both track helices. Helices projected onto the transverse plane form circles; we define *d*_m_ as the distance between the centers of the two circles minus the sum of their radii. The value of *d*_m_ is negative when the two projected trajectories intersect. We require the condition −0.25<*d*_m_<1.0 cm to be satisfied. From simulation, we have found that most of the electron–positron candidate pair background comes from misreconstructed track pairs originating from the primary vertex. These typically have negative *d*_m_ values, thus explaining the asymmetric *d*_m_ requirements.

In order to reduce the contribution of misidentified conversions from low-momentum displaced tracks that are artificially propagated back to the silicon tracker, the two candidate conversion tracks must have one of their two innermost hits in the same silicon tracker layer.

The distance along the beam line between the extrapolation of each conversion track candidate and the nearest reconstructed event vertex must be less than five times its estimated uncertainty. Moreover, among the two event vertices closest to each track along the beam line, at least one vertex must be in common

A reconstructed primary vertex is assigned to the reconstructed conversion by projecting the photon momentum onto the beamline and choosing the closest vertex along the beam direction. If the value of the distance is larger than five times its estimated uncertainty, the photon candidate is rejected.

The primary vertex associated with the conversion is required to be compatible with the reconstructed J/*ψ* vertex. This requirement is fulfilled when the three-dimensional distance between the two vertices is compatible with zero within five standard deviations. Furthermore, a check is made that neither of the two muon tracks used to define the J/*ψ* vertex is used as one of the conversion track pair.

The e^+^e^−^ track pairs surviving the selection are then fitted to a common vertex with a kinematic vertex fitter that constrains the tracks to be parallel at the vertex in both the transverse and longitudinal planes. The pair is retained if the fit *χ* ^2^ probability is greater than 0.05 %. If a track is shared among two or more reconstructed conversions, only the conversion with the larger vertex *χ* ^2^ probability is retained.

Only reconstructed conversions with transverse distance of the vertex from the center of the mean pp collision position larger than 1.5 cm are considered. This requirement suppresses backgrounds caused by track pairs originating from the primary event vertex that might mimic a conversion, such as from *π* ^0^ Dalitz decay, while retaining photon conversions occurring within the beampipe.

Finally, each conversion candidate is associated with every other conversion candidate in the event, and with any photon reconstructed using calorimeter information. Any pairs of conversions or conversion plus photon with an invariant mass between 0.11 and 0.15 GeV/*c* ^2^, corresponding to a two-standard-deviation window around the *π* ^0^ mass, is rejected. We have verified that the *π* ^0^ rejection requirement, while effectively reducing the background, does not affect the *R*_p_ measurement within its uncertainties.

Converted photon candidates are required to have *p*_T_>0.5 GeV/*c*, while no requirement is imposed on the pseudorapidity of the photon.

**Fig. 1:**
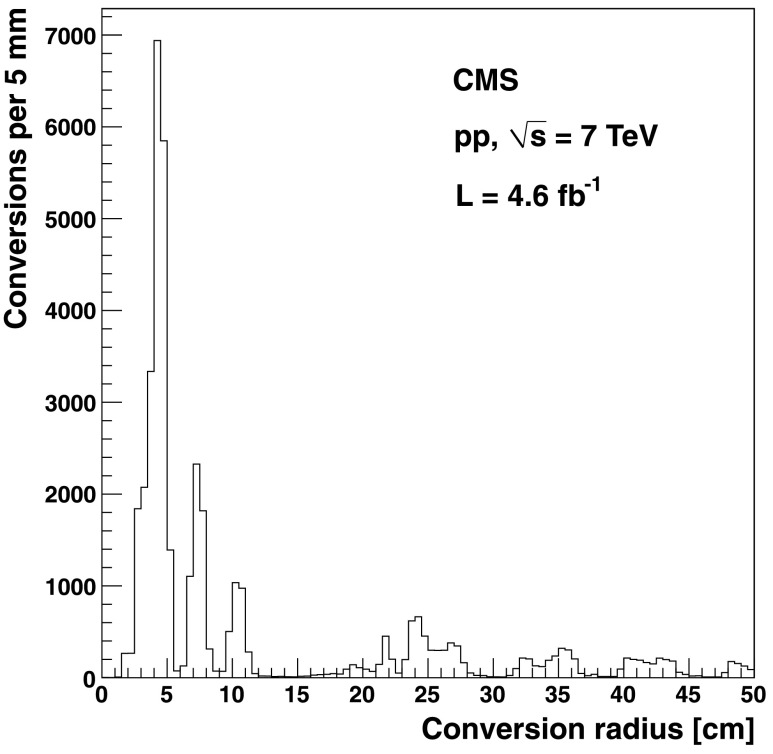
Distribution of the conversion radius for the *χ*_c_ photon candidates

The distribution of the photon conversion radius for *χ*_c_ candidates is shown in Fig. 1. The first peak corresponds to the beampipe and first pixel barrel layer, the second and third peaks correspond to the two outermost pixel layers, while the remaining features at radii larger than 20 cm are due to the four innermost silicon strip layers. The observed distribution of the photon conversion radius is consistent with the known distribution of material in the tracking volume and with Monte Carlo simulations [14].

## Acceptance and efficiencies

In the evaluation of *R*_p_, we must take into account the possibility that the geometric acceptance and the photon reconstruction efficiencies are not the same for *χ*_c1_ and *χ*_c2_.

In order to determine the acceptance correction, a Monte Carlo (MC) simulation sample of equal numbers of *χ*_c1_ and *χ*_c2_ has been used. This sample was produced using a pythia [15] single-particle simulation in which a *χ*_c1_ or *χ*_c2_ is generated with a transverse momentum distribution produced from a parameterized fit to the CMS measured *ψ*(2*S*) spectrum [16]. The use of the *ψ*(2*S*) spectrum is motivated by the proximity of the *ψ*(2*S*) mass to the states under examination. The impact of this choice is discussed in Sect. 7.

Both *χ*_c_ states in the simulation are forced to decay to J/*ψ*+*γ* isotropically in their rest frame, i.e., assuming they are produced unpolarized. We discuss later the impact of this assumption. The decay products are then processed through the full CMS detector simulation, based on Geant4 [17, 18], and subjected to the trigger emulation and the full event reconstruction. In order to produce the most realistic sample of simulated *χ*_c_ decays, digitized signals from MC-simulated inelastic pp events are mixed with those from simulated signal tracks. The number of inelastic events to mix with each signal event is sampled from a Poisson distribution to accurately reproduce the amount of pileup in the data.

The efficiency ratio *ε*_1_/*ε*_2_ for different J/*ψ* transverse momentum bins is determined using

$$\frac{\varepsilon_1}{\varepsilon_2} = \frac{N_{\chi_{\mathrm{c}1}}^\text{rec}}{N_{\chi_{\mathrm {c}1}}^\text{gen}} / \frac{N_{\chi_{\mathrm{c}2}}^\text {rec}}{N_{\chi_{\mathrm{c}2}}^\text{gen}}, $$

where *N* ^gen^ is the number of *χ*_c_ candidates generated in the MC simulation within the kinematic range |*y*(J/*ψ*)|<1.0, *p*_T_(*γ*)>0.5 GeV/*c*, and *N* ^rec^ is the number of candidates reconstructed with the selection above. The resulting values are shown in Table 1, where the uncertainties are statistical only and determined from the MC sample assuming binomial distributions. The increasing trend of *ε*_1_/*ε*_2_ is expected, because *p*_T_(J/*ψ*) is correlated with the *p*_T_ of the photon, and at higher photon *p*_T_ our conversion reconstruction efficiency is approximately constant. Therefore, efficiencies for the *χ*_c1_ and the *χ*_c2_ are approximately the same at high *p*_T_(J/*ψ*).

**Table 1:** Ratio of efficiencies *ε*_1_/*ε*_2_ as a function of the J/*ψ* transverse momentum from MC simulation. The uncertainties are statistical only

*p*_T_(J/*ψ*) [GeV/*c*]	*ε*_1_/*ε*_2_
7–9	0.903±0.023
9–11	0.935±0.019
11–13	0.945±0.021
13–16	0.917±0.022
16–20	0.981±0.031
20–25	1.028±0.049

This technique also provides an estimate of the absolute *χ*_c_ reconstruction efficiency, which is given by the product of the photon conversion probability, the *χ*_c_ selection efficiency, and, most importantly, the conversion reconstruction efficiency, which corresponds to the dominant contribution. This product varies as a function of *p*_T_(*γ*), and goes from 4×10^−4^ at 0.5 GeV/*c* to around 10^−2^ at 4 GeV/*c*, where it saturates.

## Signal extraction

We extract the numbers of *χ*_c1_ and *χ*_c2_ events, $N_{\chi_{\mathrm{c}1}}$ and $N_{\chi_{\mathrm{c}2}}$, respectively, from the data by performing an unbinned maximum-likelihood fit to the *Q* spectrum in various ranges of J/*ψ* transverse momentum.

Because of the small intrinsic width of the *χ*_c_ states we are investigating, the observed signal shape is dominated by the experimental resolution. The signal probability density function (PDF) is derived from the MC simulation described in Sect. 5, and is modeled by the superposition of two double-sided Crystal Ball functions [19] for the *χ*_c1_ and *χ*_c2_ and a single-sided Crystal Ball function for the *χ*_c0_. Each double-sided Crystal Ball function consists of a Gaussian core with exponential tails on both the high- and low-mass sides. We find this shape to provide an accurate parameterization of the *Q* spectra derived from MC simulation. When fitting the data, we fix all the parameters of the Crystal Ball function to the values that best fit our MC simulation and use a maximum-likelihood approach to derive $N_{\chi _{\mathrm{c}1}}$ and $N_{\chi_{\mathrm{c}2}}$, which are the integrals of the PDFs for the two resonances. Because the *Q* resolution depends on the *p*_T_ of the J/*ψ*, a set of shape parameters is determined for each bin of *p*_T_(J/*ψ*). Simulation shows that the most important feature of the *χ*_c0_ signal shape is the low-mass tail due to radiation from the electrons, while the high-mass tail is overwhelmed by the combinatorial background and the low-mass tail of the other resonances. Hence the choice to use a single-sided Crystal Ball function to fit the *χ*_c0_ mass distribution. Different choices of the *χ*_c0_ signal parameterization are found to cause variations in the measured *R*_p_ values that are well within the quoted systematic uncertainties given below.

The background is modeled by a probability distribution function defined as

$$N_{bkg}(Q)= (Q-q_0)^{\alpha_1} \cdot e^{(Q-q_0) \cdot\beta_1}, $$

where *α*_1_ and *β*_1_ are free parameters in the fit, and *q*_0_ is set to 3.2 GeV/*c* ^2^.

**Fig. 2:**
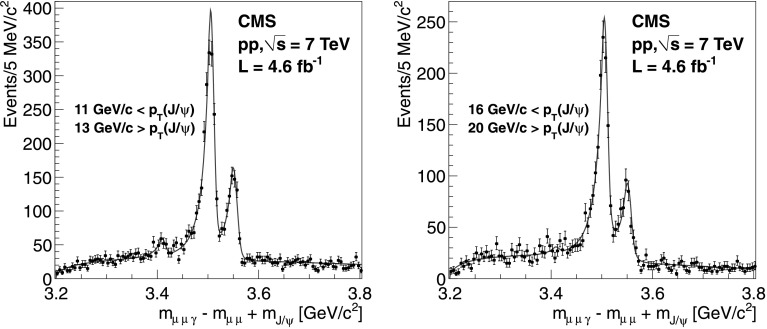
The distribution of the variable *Q*=*m*_*μμγ*_−*m*_*μμ*_+*m*_J/*ψ*_ for *χ*_*c*_ candidates with *p*_T_(J/*ψ*) ranges shown in the figures. The *line* shows the fit to the data

In Fig. 2 we show the Q distribution for two different ranges, 11<*p*_T_(J/*ψ*)<13 GeV/*c* (left) and 16<*p*_T_(J/*ψ*)<20 GeV/*c* (right). This procedure is repeated for several ranges in the transverse momentum of the J/*ψ* in order to extract $N_{\chi_{\mathrm{c}1}}$ and $N_{\chi_{\mathrm {c}2}}$ in the corresponding bin.

The results are shown in Table 2, where the reported uncertainties are statistical only.

**Table 2:** Numbers of *χ*_c1_ and *χ*_c2_ events extracted from the maximum-likelihood fit, and the ratio of the two values. Uncertainties are statistical only

*p*_T_(J/*ψ*) [GeV/*c*]	$N_{\chi_{\mathrm{c}1}}$	$N_{\chi_{\mathrm{c}2}}$	$N_{\chi_{\mathrm{c}2}} / N_{\chi_{\mathrm{c}1}}$
7–9	618 ± 31	315 ± 24	0.510 ± 0.049
9–11	1680 ± 49	788 ± 37	0.469 ± 0.027
11–13	1819 ± 51	819 ± 38	0.451 ± 0.025
13–16	1767 ± 51	851 ± 39	0.482 ± 0.027
16–20	1269 ± 43	487 ± 30	0.384 ± 0.028
20–25	642 ± 31	236 ± 22	0.368 ± 0.040

## Systematic uncertainties

Several types of systematic uncertainty are addressed. In particular, we investigate possible effects that could influence the measurement of the numbers of *χ*_c1_ and *χ*_c2_ from data, the evaluation of *ε*_1_/*ε*_2_ from the MC simulation, and the derivation of the *R*_p_ ratio. In Table 3 the various sources of systematic uncertainties and their contributions to the total uncertainty are summarized. The following subsections describe how the various contributions are evaluated.

**Table 3:** Relative systematic uncertainties on *R*_p_ for different ranges of J/*ψ* transverse momentum from different sources and the total uncertainty

*p*_T_(J/*ψ*) range [GeV/*c*]	7–9	9–11	11–13	13–16	16–20	20–25
Source of uncertainty	Systematic uncertainty (%)					
Background shape	1.4	1.5	0.9	1.2	1.8	2.4
Signal shape	1.4	3.0	1.1	1.5	1.5	2.3
Simulation sample size	2.6	2.0	2.2	2.4	3.1	4.8
Choice of *p*_T_(*χ*_c_) spectrum	4.5	3.7	2.9	1.9	0.6	1.1
Total uncertainty	5.5	5.4	3.9	3.6	4.0	5.9

### Uncertainty from the mass fit and *χ*_c1_ and *χ*_c2_ counting

The measurement of the ratio $N_{\chi_{\mathrm{c}2}}/N_{\chi_{\mathrm{c}1}}$ could be affected by the choice of the functional form used for the maximum-likelihood fit. The use of an alternative background parameterization, a fourth-order polynomial, results in systematically higher values of the ratio $N_{\chi_{\mathrm {c}2}}/N_{\chi_{\mathrm{c}1}}$, while keeping the overall fit quality as high as in the default procedure. From the difference in the numbers of signal events using the two background parameterizations, we assign the systematic uncertainty from the background modeling shown in Table 3.

We evaluate the systematic uncertainty related to the parameterization of the signal shape by varying the parameters derived from the MC simulation within their uncertainties. The results fluctuate within 1–3 % in the various transverse momentum ranges. We assign the systematic uncertainties from this source, as shown in Table 3.

The method to disentangle and count the *χ*_c1_ and *χ*_c2_ states is validated by using a pythia MC simulation sample of inclusive J/*ψ* events, including those from *χ*_c_ decay, produced in pp collisions and propagated through the full simulation of the detector. The ratio $N_{\chi _{\mathrm{c}2}}/N_{\chi_{\mathrm{c}1}}$ derived from the fit to the Q distribution of the reconstructed candidates in the simulation is consistent with the actual number of *χ*_c_ events contributing to the distribution, within the statistical uncertainty, for all J/*ψ* momentum ranges. Therefore, we do not assign any further systematic uncertainty in the determination of $N_{\chi_{\mathrm{c}2}}/N_{\chi_{\mathrm{c}1}}$.

The stability of our analysis as a function of the number of primary vertices in the event has been investigated. The number of *χ*_c_ candidates per unit of integrated luminosity, once trigger conditions are taken into account, is found to be independent of the instantaneous luminosity, within the statistical uncertainties. In addition, the measured ratio $N_{\chi_{\mathrm{c}2}}/N_{\chi _{\mathrm{c}1}}$ is found to be constant as a function of the number of primary vertices in the event, within the statistical uncertainties. Thus, no systematic uncertainty due to pileup is included in the final results.

### Uncertainty in the ratio of efficiencies

The statistical uncertainty in the measurement of *ε*_1_/*ε*_2_ from the simulation, owing to the finite size of the MC sample, is taken as a systematic uncertainty, as shown in Table 3.

Since the analysis relies on photon conversions, the effect of a possible incorrect simulation of the tracker detector material is estimated. Two modified material scenarios, i.e., special detector geometries prepared for this purpose, in which the total mass of the silicon tracker varies by up to 5 % from the reference geometry, are used to produce new MC simulation samples [20]. With these models, local variations of the radiation length with respect to the reference simulation can be as large as +8 % and −3 %. No significant difference in the ratio of efficiencies is observed and the corresponding systematic uncertainty is taken to be negligible.

Several choices of the generated *p*_T_(*χ*_c_) spectrum are investigated. In particular, the use of the measured J/*ψ* spectrum [11] gives values that are compatible with the default *ψ*(2*S*) spectrum used for the final result. The choice of the spectrum affects the values of *ε*_1_/*ε*_2_ only inasmuch as we perform an average measurement in each bin of *p*_T_(J/*ψ*), and the size of these bins is finite. We choose to assign a conservative systematic uncertainty by comparing the values of *ε*_1_/*ε*_2_ obtained with the *ψ*(2*S*) spectrum with those obtained in the case where the *p*_T_(*χ*_c_) spectrum is taken to be constant in each *p*_T_ bin. The corresponding systematic uncertainties are given in Table 3.

### *χ*_*c*_ polarization

The polarizations of the *χ*_c1_ and *χ*_c2_ are unknown. Efficiencies are estimated under the assumption that the two states are unpolarized. If the *χ*_c_ states are polarized, the resulting photon angular distribution and transverse momentum distributions will be affected. This can produce a change in the photon efficiency ratio *ε*_1_/*ε*_2_.

In order to investigate the impact of different polarization scenarios on the ratio of the efficiencies, we reweight the unpolarized MC distributions to reproduce the theoretical *χ*_c_ angular distributions [21, 22] for different *χ*_c_ polarizations. We measure the efficiency *ε*_1_/*ε*_2_ for the *χ*_c1_ being unpolarized or with helicity $m_{\chi_{\mathrm{c}1}} =0,\pm1$, in combination with the *χ*_c2_ being unpolarized or having helicity $m_{\chi _{\mathrm{c}2}} =0, \pm2 $ in both the helicity and Collins–Soper [23] frames. The ratio of efficiencies for the cases involving $m_{\chi_{\mathrm{c}2}} = \pm1 $ is between the cases with $m_{\chi_{\mathrm{c}2}} = 0 $ and $m_{\chi_{\mathrm {c}2}} = \pm2 $. Tables 4 and 5 give the resulting *ε*_1_/*ε*_2_ values for each polarization scenario in different J/*ψ* transverse momentum bins for the two frames, relative to the value of the ratio for the unpolarized case. These tables, therefore, provide the correction that should be applied to the default value of *ε*_1_/*ε*_2_ in each polarization scenario and each range of transverse momentum.

**Table 4:** The efficiency ratio *ε*_1_/*ε*_2_ for different polarization scenarios in which the *χ*_c1_ is either unpolarized or has helicity $m_{\chi_{c1}}=0,\pm1$ and the *χ*_c2_ is either unpolarized or has helicity $m_{\chi_{c2}}=0,\pm2$ in the helicity frame, relative to the unpolarized case

Polarization scenario ($m_{\chi_{c1}},m_{\chi_{c2}}$)	*p*_T_(J/*ψ*) [GeV/*c*]					
	7–9	9–11	11–13	13–16	16–20	20–25
(Unpolarized,0)	0.89	0.87	0.85	0.86	0.85	0.86
(Unpolarized,±2)	1.20	1.20	1.21	1.20	1.20	1.17
(0,Unpolarized)	0.83	0.84	0.85	0.85	0.85	0.86
(±1,Unpolarized)	1.08	1.07	1.07	1.07	1.07	1.07
(0,0)	0.74	0.73	0.72	0.73	0.72	0.74
(0,±2)	1.00	1.01	1.03	1.02	1.02	1.01
(±1,0)	0.95	0.93	0.91	0.97	0.90	0.92
(±1,±2)	1.29	1.29	1.29	1.28	1.28	1.25

**Table 5:** The values of *ε*_1_/*ε*_2_ for different polarization scenarios in the Collins–Soper frame, relative to the unpolarized case

Polarization scenario ($m_{\chi_{c1}},m_{\chi_{c2}}$)	*p*_T_(J/*ψ*) [GeV/*c*]					
	7–9	9–11	11–13	13–16	16–20	20–25
(Unpolarized,0)	1.04	1.06	1.08	1.07	1.08	1.08
(Unpolarized,±2)	0.97	0.95	0.93	0.93	0.92	0.92
(0,Unpolarized)	1.04	1.05	1.06	1.07	1.07	1.06
(±1,Unpolarized)	0.98	0.97	0.97	0.96	0.96	0.97
(0,0)	1.08	1.12	1.14	1.15	1.16	1.14
(0,±2)	1.01	0.99	0.98	0.99	0.98	0.98
(±1,0)	1.02	1.03	1.04	1.04	1.04	1.04
(±1,±2)	0.95	0.92	0.90	0.90	0.89	0.89

### Branching fractions

The measurement of the prompt *χ*_c2_ to *χ*_c1_ production cross section ratio is affected by the uncertainties in the branching fractions of the two states into J/*ψ*+*γ*. The quantity that is directly accessible in this analysis is *R*_p_, the product of the ratio of the *χ*_c2_ to *χ*_c1_ cross sections and the ratio of the branching fractions.

In order to extract the ratio of the prompt production cross sections, we use the value of 1.76±0.10 for $\mathcal{B}(\chi_{\mathrm {c}1}\to \mathrm{J}/\psi+ \gamma) / \mathcal{B}(\chi_{\mathrm {c}2}\to \mathrm{J}/\psi + \gamma)$ as derived from the branching fractions and associated uncertainties reported in Ref. [9].

## Results and discussion

**Fig. 3:**
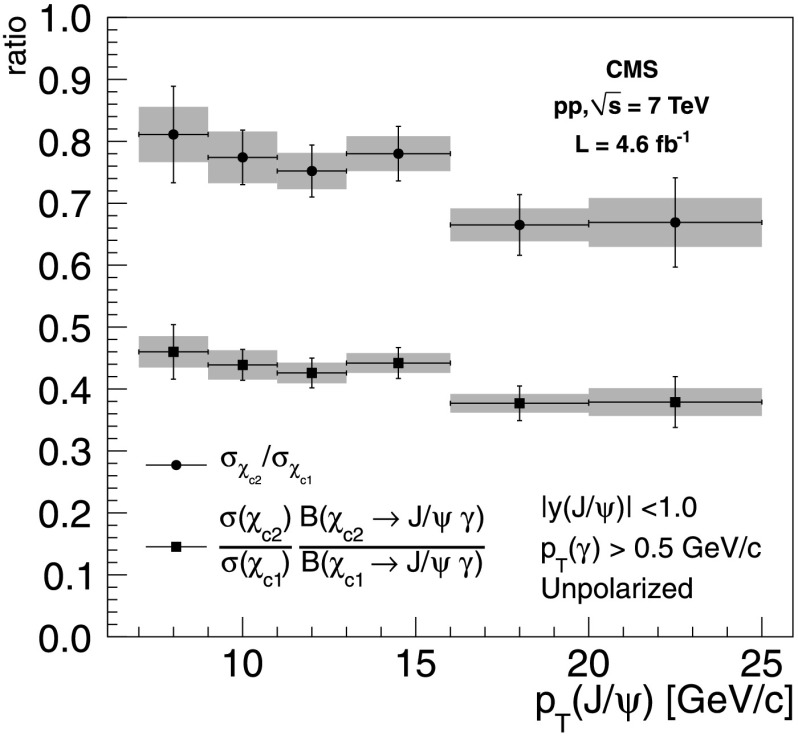
Ratio of the *χ*_c2_ to *χ*_c1_ production cross sections (*circles*) and ratio of the cross sections times the branching fractions to J/*ψ*+*γ* (*squares*) as a function of the J/*ψ* transverse momentum with the hypothesis of unpolarized production. The *error bars* correspond to the statistical uncertainties and the *band* corresponds to the systematic uncertainties. For the cross section ratios, the 5.6 % uncertainty from the branching fractions is not included

The results of the measurement of the ratio *R*_p_ and of the ratio of the *χ*_c2_ to *χ*_c1_ prompt production cross sections for the kinematic range *p*_T_(*γ*)>0.5 GeV/*c* and |*y*(J/*ψ*)|<1.0 are reported in Tables 6 and 7, respectively, for different ranges of *p*_T_(J/*ψ*). The first uncertainty is statistical, the second is systematic, and the third comes from the uncertainty in the branching fractions in the measurement of the cross section ratio. Separate columns are dedicated to the uncertainty derived from the extreme polarization scenarios in the helicity and Collins–Soper frames, by choosing from Tables 4 and 5 the scenarios that give the largest variations relative to the unpolarized case. These correspond to $(m_{\chi_{\mathrm{c}1}}, m_{\chi_{\mathrm {c}2}}) = (\pm1,\pm2)$ and $(m_{\chi_{\mathrm{c}1}}, m_{\chi _{\mathrm{c}2}}) = (0,0)$ for both the helicity and Collins–Soper frames. Figure 3 displays the results as a function of the J/*ψ* transverse momentum for the hypothesis of unpolarized production. The error bars represent the statistical uncertainties and the green bands the systematic uncertainties.

**Table 6:** Measurements of $\frac{ \sigma({\chi_{\mathrm {c}2}})\mathcal{B}(\chi_{\mathrm{c}2})}{ \sigma({\chi_{\mathrm {c}1}})\mathcal{B}(\chi_{\mathrm{c}1})}$ for the given *p*_T_(J/*ψ*) ranges in the fiducial kinematic region *p*_T_(*γ*)>0.5 GeV/*c*, |*y*(J/*ψ*)|<1.0, assuming unpolarized *χ*_c_ production. The first uncertainty is statistical and the second is systematic. The last two columns report the additional uncertainties derived from the extreme polarization scenarios in the helicity (HX) and Collins–Soper (CS) frames

*p*_T_(J/*ψ*) [GeV/*c*]	$\frac{ \sigma({\chi_{\mathrm{c}2}})\mathcal{B}(\chi_{\mathrm {c}2})}{\sigma({\chi_{\mathrm{c}1}})\mathcal{B}(\chi_{\mathrm{c}1})}$	HX	CS
7–9	0.460±0.044 (stat.) ± 0.025 (syst.)	${}^{+0.136}_{-0.121}$	${}^{+0.037}_{-0.023}$
9–11	0.439±0.025 (stat.) ± 0.024 (syst.)	${}^{+0.128}_{-0.119}$	${}^{+0.052}_{-0.035}$
11–13	0.426±0.024 (stat.) ± 0.017 (syst.)	${}^{+0.125}_{-0.117}$	${}^{+0.059}_{-0.042}$
13–16	0.442±0.025 (stat.) ± 0.016 (syst.)	${}^{+0.125}_{-0.121}$	${}^{+0.065}_{-0.044}$
16–20	0.377±0.028 (stat.) ± 0.015 (syst.)	${}^{+0.106}_{-0.104}$	${}^{+0.059}_{-0.042}$
20–25	0.379±0.041 (stat.) ± 0.022 (syst.)	${}^{+0.094}_{-0.097}$	${}^{+0.055}_{-0.040}$

**Table 7:** Measurements of *σ*(*χ*_c2_)/*σ*(*χ*_c1_) for the given *p*_T_(J/*ψ*) ranges derived using the branching fractions from Ref. [9], assuming unpolarized *χ*_c_ production. The first uncertainty is statistical, the second is systematic, and the third from the branching fraction uncertainties. The last two columns report the uncertainties derived from the extreme polarization scenarios in the helicity (HX) and Collins–Soper (CS) frames

*p*_T_(J/*ψ*) [GeV/*c*]	*σ*(*χ*_c2_)/*σ*(*χ*_c1_)	HX	CS
7–9	0.811±0.078 (stat.) ± 0.045 (syst.) ± 0.046(BR)	${}^{+0.239}_{-0.213}$	${}^{+0.066}_{-0.041}$
9–11	0.774±0.044 (stat.) ± 0.042 (syst.) ± 0.044(BR)	${}^{+0.225}_{-0.209}$	${}^{+0.092}_{-0.061}$
11–13	0.752±0.042 (stat.) ± 0.029 (syst.) ± 0.043(BR)	${}^{+0.221}_{-0.207}$	${}^{+0.105}_{-0.074}$
13–16	0.78±0.044 (stat.) ± 0.028 (syst.) ± 0.044(BR)	${}^{+0.221}_{-0.213}$	${}^{+0.115}_{-0.078}$
16–20	0.665±0.049 (stat.) ± 0.027 (syst.) ± 0.038(BR)	${}^{+0.187}_{-0.184}$	${}^{+0.104}_{-0.074}$
20–25	0.669±0.072 (stat.) ± 0.039 (syst.) ± 0.038(BR)	${}^{+0.165}_{-0.172}$	${}^{+0.096}_{-0.070}$

Our measurement of the ratio of the prompt *χ*_c2_ to *χ*_c1_ cross sections includes both directly produced *χ*_c_ mesons and indirectly produced ones from the decays of intermediate states. To convert our result to the ratio of directly produced *χ*_c2_ to *χ*_c1_ mesons requires knowledge of the amount of feed-down from all possible short-lived intermediate states that have a decay mode into *χ*_c2_ or *χ*_c1_. The largest known such feed-down contribution comes from the *ψ*(2*S*). Using the measured prompt J/*ψ* and *ψ*(2*S*) cross sections in pp collisions at 7 TeV [16], the branching fractions for the decays *ψ*(2*S*)→*χ*_c1,2_+*γ* [9], and assuming the same fractional *χ*_c_ contribution to the total prompt J/*ψ* production cross section as measured in $\mathrm{p}\mathrm{\overline {p}}$ collisions at 1.96 TeV [24], we estimate that roughly 5 % of both our prompt *χ*_c1_ and *χ*_c2_ samples come from *ψ*(2*S*) decays. The correction in going from the prompt ratio to the direct ratio is about 1 %. In comparing our results with the theoretical predictions described below, we have not attempted to correct for this effect since the uncertainties on the fractions are difficult to estimate, the correction is much smaller than the statistical and systematic uncertainties, and our conclusions on the comparisons with the theoretical predictions would not be altered by a correction of this magnitude.

**Fig. 4:**
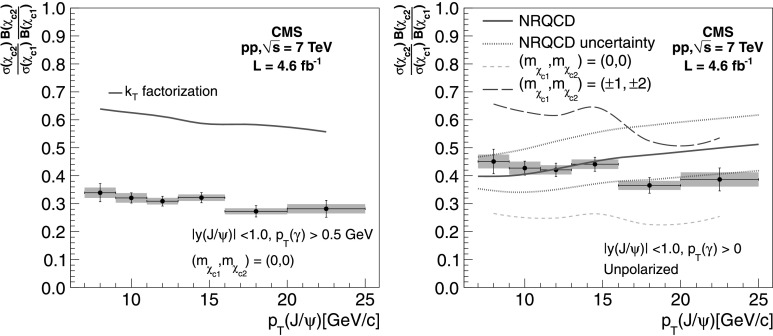
Comparison of the measured $\frac{\sigma({\chi_{\mathrm {c}2}})\mathcal{B}(\chi_{\mathrm{c}2}) }{ \sigma({\chi_{\mathrm {c}1}})\mathcal{B}(\chi_{\mathrm{c}1})}$ values with theoretical predictions from the *k*_T_-factorization [6] (*left*) and NRQCD [7] (*right*) calculations (*solid red lines*). The *error bars* and *bands* show the experimental statistical and systematic uncertainties, respectively. The measurements *in the left plot* use an acceptance correction assuming zero helicity for the *χ*_c_, as predicted by the *k*_T_-factorization model. The measurements *in the right plot* are corrected to match the kinematic range used in the NRQCD calculation and assume the *χ*_c_ are produced unpolarized. The measurements assuming two different extreme polarization scenarios are shown by the *long-dashed* and *short-dashed* lines *in the plot on the right*. The 1-standard-deviation uncertainties in the NRQCD prediction, originating from uncertainties in the color-octet matrix elements, are displayed as the *dotted lines*

We compare our results with theoretical predictions derived from the *k*_T_-factorization [6] and NRQCD [7] calculations in Fig. 4. The *k*_T_-factorization approach predicts that both *χ*_c1_ and *χ*_c2_ are produced in an almost pure helicity-zero state in the helicity frame. Therefore, in our comparison, we apply the corresponding correction on the ratio of efficiencies from Table 4, amounting to a factor of 0.73, almost independent of *p*_T_. The theoretical calculation is given in the same kinematic range (*p*_T_(*γ*)>0.5 GeV/*c*, |*y*(J/*ψ*)|<1.0) as our measurement. There is no information about the *χ*_c_ polarization from the NRQCD calculations, so we use the ratio of efficiencies estimated in the unpolarized case for our comparison. The prediction is given in the kinematic range *p*_T_(*γ*)>0 GeV/*c*, |*y*(J/*ψ*)|<1.0. We use the same MC simulation described in Sect. 5 to derive the small correction factor (ranging from 0.98 to 1.02 depending on *p*_T_, with uncertainties from 1 to 4 %) needed to extrapolate the phase space of our measurement to the one used for the theoretical calculation. The uncertainty in the correction factor stemming from the assumption of the *χ*_c_ transverse momentum distribution is added as a systematic uncertainty. The values of *R*_p_ after extrapolation are shown in Table 8. The comparison of our measurements with the *k*_T_-factorization and NRQCD predictions are shown in the left and right plots of Fig. 4, respectively. The *k*_T_-factorization prediction agrees well with the trend of *R*_p_ versus transverse momentum of the J/*ψ*, but with a global normalization that is higher by about a factor two with respect to our measurement. It is worth noting that this calculation assumes the same wave function for the *χ*_c1_ and the *χ*_c2_. On the other hand, the NRQCD prediction is compatible with our results within the experimental and theoretical uncertainties, though, since predictions for *χ*_c1_ or *χ*_c2_ polarizations were not provided, the level of agreement can vary considerably.

**Table 8:** Measurements of $\frac{ \sigma({\chi_{\mathrm {c}2}})\mathcal{B}(\chi_{\mathrm{c}2})}{ \sigma({\chi_{\mathrm {c}1}})\mathcal{ B}(\chi_{\mathrm{c}1})}$ for the given *p*_T_(J/*ψ*) ranges after extrapolating the measurement to the kinematic region *p*_T_(*γ*)>0 and assuming unpolarized *χ*_c_ production. The first uncertainty is statistical and the second is systematic. The last column reports the largest variations due changes in the assumed *χ*_c_ polarizations

*p*_T_(J/*ψ*) [GeV/*c*]	$\frac{ \sigma({\chi_{\mathrm{c}2}})\mathcal{B}(\chi_{\mathrm {c}2})}{ \sigma({\chi_{\mathrm{c}1}})\mathcal{B}(\chi_{\mathrm {c}1})}$	Polarization
7–9	0.451 ± 0.043 (stat.) ± 0.025 (syst.)	${}^{+ 0.137} _{- 0.153}$
9–11	0.427 ± 0.024 (stat.) ± 0.023 (syst.)	${}^{+ 0.134} _{- 0.144}$
11–13	0.421 ± 0.024 (stat.) ± 0.017 (syst.)	${}^{+ 0.133} _{- 0.142}$
13–16	0.441 ± 0.025 (stat.) ± 0.017 (syst.)	${}^{+ 0.138} _{- 0.143}$
16–20	0.365 ± 0.027 (stat.) ± 0.016 (syst.)	${}^{+ 0.114} _{- 0.115}$
20–25	0.387 ± 0.042 (stat.) ± 0.026 (syst.)	${}^{+ 0.109} _{- 0.105}$

A direct comparison of our results with previous measurements, in particular from [4] and [5], is not straightforward, because of the different conditions under which they were carried out. Specifically, there are differences in the kinematical phase space considered and, in the case of [4], in the initial-state colliding beams and center-of-mass energy used. However, with these caveats, a direct comparison shows that the three results are compatible within their uncertainties. In particular, all three results confirm the trend of a decreasing ratio of *χ*_c2_ to *χ*_c1_ production cross sections as a function of *p*_T_(J/*ψ*), under the assumption that the *χ*_c2_ and *χ*_c1_ polarizations do not depend on *p*_T_(J/*ψ*).

## Summary

Measurements have been presented of the ratio

$$R_\mathrm{p} \equiv \frac{\sigma(\mathrm{p}\mathrm{p}\to\chi_{\mathrm{c}2}+X ) \mathcal{B}(\chi_{\mathrm{c}2}\to{\mathrm{J}/\psi}+ \gamma) }{ \sigma(\mathrm{p}\mathrm{p}\to\chi_{\mathrm{c}1}+X ) \mathcal {B}(\chi_{\mathrm{c}1}\to{\mathrm{J}/\psi}+ \gamma) } $$

as a function of the J/*ψ* transverse momentum up to ${p_{\mathrm{T}}}({\mathrm{J}/\psi}) = 25~\text {GeV$/c$}$ for the kinematic range *p*_T_(*γ*)>0.5 GeV/*c* and |*y*(J/*ψ*)|<1.0 in pp collisions at $\sqrt{s} = 7~\text{TeV}$ with a data sample corresponding to an integrated luminosity of 4.6 fb^−1^. The corresponding values for the ratio of the *χ*_c2_ to *χ*_c1_ production cross sections have been determined.

The results have also been shown after extrapolating the photon acceptance down to zero *p*_T_. The effect of several different *χ*_c_ polarization scenarios on the photon reconstruction efficiency has been investigated and taken into account in the comparison of the experimental results with two recent theoretical predictions. This is among the most precise measurements of the *χ*_c_ production cross section ratio made in hadron collisions, and extends the explored J/*ψp*_T_ range of previous results. These measurements will provide important input to and constraints on future theoretical calculations of quarkonium production, as recently discussed in [25] for the bottomonium family.
